# Impact of Digitalization on Performance of Dutch Hospitals: Longitudinal Quantitative Study

**DOI:** 10.2196/94896

**Published:** 2026-09-29

**Authors:** Philipp Remus, Daan Westra, Rachel Gifford, Frank van de Baan, Mark Govers

**Affiliations:** 1 Department of Health Services Research Care and Public Health Research Institute (CAPHRI) Maastricht University Maastricht, Limburg The Netherlands

**Keywords:** digital maturity, digital transformation, electronic health records, hospital performance, maturity models, productivity paradox

## Abstract

**Background:**

Hospitals continue to invest heavily to increase their level of digitalization. While advanced digital maturity is assumed to improve hospital performance, empirical evidence remains mixed. This tension is mirrored by the productivity paradox of IT, whereby investments in digital technologies do not consistently translate into observable performance gains.

**Objective:**

This study aims to examine the relationship between the Healthcare Information and Management Systems Society (HIMSS) Electronic Medical Record Adoption Model (EMRAM) score and hospitals’ financial, operational, and workforce-related indicators.

**Methods:**

This longitudinal observational study used routinely collected hospital-level annual report data from the Dutch National Annual Healthcare Reports Database (CIBG) for Dutch hospitals from 2017 to 2023. Hospital-year records were linked at the institutional level to publicly available HIMSS EMRAM stage 6 or 7 certification information and operationalized dichotomously (stage 6 or 7 vs ≤5). The sample included 66 to 74 hospitals per year (mean 70.4, SD 2.8), corresponding to up to 498 hospital-year observations. Outcome measures were financial (profit margins, return on assets, asset-turnover ratio, and personnel-expense ratio), operational (length of stay and number of patients treated), and workforce (absenteeism) performance indicators. Linear mixed-effects models were estimated while controlling for hospital size, teaching status, staff-to-patient ratio, time trends, and COVID-19 effects.

**Results:**

Advanced digital maturity was not significantly associated with improved financial, operational, or workforce performance after adjustment for multiple testing. For financial outcomes, high digital maturity showed no significant association with profit margin, return on assets, personnel-expense ratio, or asset-turnover ratio. Digitally mature hospitals initially appeared to treat more patients annually (*β*=31,664.318; 95% CI 8392.095-54,936.540; *P*=.008), but this association was not statistically significant after Holm-Bonferroni correction (adjusted *P*=.44). No significant associations were observed for length of stay or absenteeism.

**Conclusions:**

In a highly digitalized health system with near-universal electronic health record adoption, advanced technical digital maturity alone was not associated with measurable improvements in aggregated hospital-level financial, operational, or workforce performance. These findings provide longitudinal empirical support for the IT productivity paradox in hospital digitalization, suggesting that technical maturity is a necessary but insufficient condition for performance gains.

## Introduction

The health care sector is undergoing a major paradigm shift toward intensive digitalization, a trend significantly accelerated by the COVID-19 pandemic [1,2]. This shift is characterized by the integration of advanced technologies such as electronic health records (EHRs) [3], blockchain [4], big data [5], and AI [6]. Despite the rapid developments in digitalization in the health care sector, empirical evidence on how digital maturity translates into measurable hospital performance remains limited and fragmented. Previous studies have focused on the adoption phase of EHRs, emphasizing implementation barriers [7,8] and early outcomes such as efficiency, safety, and experience [9-11], rather than the overall impact of advanced digital maturity on hospital performance. As health systems move beyond the initial adoption phase, the question shifts from whether hospitals implement digital systems to whether higher levels of digital maturity translate into measurable organizational value. Understanding whether digitally advanced hospitals outperform their peers is relevant for investment priorities and strategy, as well as for informing national digital health strategies.

When digital maturity models are used, studies frequently use cross-sectional designs [10,12-14], which limits the temporal understanding of digital capabilities and their effect on organizational performance [15]. Because financial, operational, and workforce improvements are shaped by managerial decisions and organizational processes, it remains unclear whether hospitals that achieve higher Electronic Medical Record Adoption Model (EMRAM; see Independent Variables section) stages realize improvements. At the same time, advanced digital maturity is often expected to enhance coordination, streamline workflows, and support more efficient resource use [16,17]. However, these expected benefits depend on successful organizational integration, workflow redesign, and user adaptation [18,19]. This tension between high expectations and inconsistent empirical evidence mirrors the well-documented productivity paradox of information technology [20-23], in which substantial digital investments do not necessarily yield observable performance gains [22,23].

The present study addresses the gap by providing longitudinal, multiyear evidence on the relationship between hospitals’ digital maturity and their financial, operational, and workforce performance over a 7-year period in the Netherlands. Given the mixed empirical evidence on the performance effects of advanced digitalization and considerations related to the IT productivity paradox, we formulate 2 hypotheses. The productivity paradox literature suggests that digital investments do not always translate into measurable performance improvements, particularly when complementary organizational changes do not accompany technological capabilities. However, advanced digital maturity is expected to enhance information availability, improve care coordination, and streamline clinical and administrative workflows, which may ultimately lead to efficiency gains and improved organizational performance [16,24,25]. Building on prior evidence linking advanced digitalization to improved efficiency [9] and care quality [10], we hypothesize that hospitals with a high degree of digital maturity will demonstrate comparatively higher financial and operational performance, reflected in their profit margins, return on assets (ROA), and asset turnover ratios, as well as patient throughput and patients’ length of stay (hypothesis 1). Additionally, it is hypothesized that higher digital maturity may be associated with improved workforce outcomes, illustrated by reduced absenteeism, given the potential of digital systems to streamline workflows and reduce the administrative burden (hypothesis 2).

## Methods

### Overview

This longitudinal study, spanning over 7 years (2017-2023), uses a quantitative research design based on annual report data made publicly available by the Dutch Ministry of Health to investigate the impact of digitalization on hospitals’ performance. A linear mixed-effects model was used to examine the relationship between the degree of digitalization and hospitals’ performance.

### Setting

This study was conducted in the Netherlands, where hospitals and independent treatment centers provide curative care [26]. Dutch hospitals operate as private, nonprofit entities that engage in annual contractual agreements with private health insurers, which are the primary funding source [27]. The reimbursement model follows a diagnosis-related group (DRG)–like funding system [27]. In 2023, around 70 hospitals were active in the Netherlands, 8 of which were academic hospitals [28]. Academic hospitals differ from general hospitals as they are allowed to provide more specialized care and contribute significantly to research and education [26].

The Dutch hospital sector is characterized by a relatively high baseline level of digital adoption, with longstanding investments in electronic health records and health information exchange, leading to a 97% EHR adoption rate among clinicians compared with a European average of 81% [2]. The Netherlands also performs favorably on several other digital infrastructure indicators, including electronic prescribing, online appointment booking, apps for clinicians, telemedicine, and automation of pharmacy drug dispensing, while still exhibiting variation in the extent to which advanced digital capabilities are realized across hospitals [2]. Hence, digital transformation is increasingly treated as an organizational and strategic issue in Dutch hospitals [29]. Recent qualitative evidence found that all participating hospitals had a team or a program dedicated to digital transformation, although they differed in focus and governance structure [29].

### Data and Sample

This study used a publicly available national dataset of annual report data from Dutch health care organizations from the Dutch National Annual Healthcare Reports Database (CIBG). This dataset includes mandatory annual data from all hospitals in the Netherlands, comprising financial (eg, revenue, profit, assets, and liabilities) and operational (eg, number of beds, personnel, and admissions) indicators [28,30]. The study population was selected at the institutional level. All Dutch hospitals with available CIBG annual-report data between 2017 and 2023 were eligible for inclusion. Nonhospital health care organizations and independent treatment centers were not included in the sample. Hospital-year observations were retained when the variables required for a given outcome model were available. CIBG hospital-year records were linked at the institutional level to publicly available information on Healthcare Information and Management Systems Society (HIMSS) EMRAM stage 6 or 7 certification. Linkage was based on hospital name, organizational identity, and manual verification of publicly available announcements. To assess hospitals’ digital maturity, this study used HIMSS EMRAM, a widely used and internationally recognized benchmarking framework for hospital digitalization [31,32]. HIMSS EMRAM is consistently applied across health systems and countries and is based on a standardized assessment and validation process conducted by HIMSS, enabling longitudinal and cross-hospital comparability [12,33,34]. Although EMRAM is not a direct measure of organizational transformation or digital value realization, its standardized HIMSS validation process supports its use as a reliable external indicator of advanced electronic medical record capability and cross-hospital comparability. For these reasons, EMRAM provides an appropriate and operationally feasible proxy for assessing hospitals’ digital maturity, particularly when the study aims to examine the relationship between technological maturity and measurable performance outcomes such as efficiency, financial results, and quality of care. All hospitals for which data were available in the CIBG dataset were included. Across all variables and years, approximately 1.63% (n=65) hospital-year observations were missing. When data for a given hospital year were missing, efforts were made to retrieve them from alternative public records (eg, archived reports). If data could not be obtained, the hospital was excluded only for the affected variable, resulting in variable-specific sample sizes. Missing data were handled via available-case deletion. Missingness was limited, largely unrelated to hospital characteristics, and concentrated in derived financial ratios for which imputation may produce implausible or inconsistent values. Because the mixed-effects framework allows variable-specific sample sizes, available-case deletion provided a transparent and conservative approach [35].

The final sample included between 66 and 74 hospitals per year (mean 70.4, SD 2.8). The longitudinal structure thus comprised up to 498 hospital-year observations across the 7-year period (2017-2023).

### Variables

This analysis focuses on hospital performance using a multidimensional organizational-level framework, distinguishing between financial performance, operational efficiency, and workforce outcomes. The selected indicators reflected standardized, routinely reported hospital metrics [36] that enable longitudinal comparison. An operationalization table of the outcome variables is provided in Table 1.

**Table 1:** Overview of dependent variables used to measure hospital performance.

Category and outcome variable		Definition/formula	Concept measured	Expected direction with higher digital maturity (HIMSS^a^ EMRAM^b^ stage 6 or 7)
**Financial performance**				
	Profit margin	Net income/total revenue	Measures hospital profitability and financial efficiency	↑ Higher digital maturity is expected to improve financial efficiency
	Return on assets	Results after taxes/total assets	Captures how effectively hospitals use assets to generate profit	↑ Improved asset usage through digital integration
	Personnel-expense ratio	Personnel costs/operating revenue	Reflects cost efficiency in relation to staffing expenditures	↓ Expected reduction through workflow efficiency
	Asset-turnover ratio	Total revenue/total assets	Indicates how efficiently assets generate revenue	↑ Increased asset productivity via digitalized processes
**Operational efficiency**				
	Number of patients treated	Total annual inpatient and outpatient cases	Represents hospital throughput and service capacity	↑ Higher throughput because of streamlined digital workflows
	Length of stay	Total inpatient days/number of discharges	Reflects process efficiency and resource usage	↓ Expected shorter stays through process optimization
**Workforce outcome**				
	Absenteeism rate	Percentage of total staff absent (sick leave) per year	Indicates workforce well-being and organizational health	↓ Improved working conditions due to digital support tools

^a^HIMSS: Healthcare Information and Management Systems Society.

^b^EMRAM: Electronic Medical Record Adoption Model.

### Dependent Variables

Financial performance was measured using profit margins [7,37,38], ROA [3,39], personnel-expense ratio [40,41], and asset-turnover ratio [42,43], commonly used indicators of hospitals’ financial performance [40,43]. Given the study’s focus on longitudinal comparability and the use of standardized, routinely reported data, operational efficiency is intentionally conceptualized in a narrower, throughput-oriented, and usage-oriented sense. Operational efficiency was measured through the number of patients treated and the length of stay, reflecting service throughput and usage. The number of patients treated represents the total annual inpatient and outpatient volume [28] and serves as an indicator of a hospital’s service-delivery capacity and operational throughput [44]. It reflects the extent to which hospitals can process patient demand within existing infrastructure and staffing constraints. The length of stay was calculated as the ratio of total inpatient days to the number of discharges [41,45]. While length of stay is influenced by case mix and clinical complexity, at the aggregate hospital level, it is considered to identify resource use [46]. As a workforce outcome, the absenteeism rate was measured, defined as the percentage of staff absent due to sick leave [28,47]. Absenteeism is commonly used as an indicator of workforce well-being, organizational strain, and operational stability [48]. At the hospital level, it provides insight into whether digital transformation is associated with improved or deteriorating working conditions over time [49].

### Independent Variables

In health care, digital maturity refers to the extent to which a hospital effectively leverages digital technologies to support high-quality care delivery, resulting in improved services and service delivery for an enhanced patient experience [50]. Importantly, digital maturity goes beyond the mere adoption of health information technologies [51,52]. Rather, it reflects how well digital systems are embedded in clinical and administrative processes and how they are used to generate organizational value over time [53]. Conceptually, digital maturity denotes a state of organizational readiness and capability that evolves in response to technological, organizational, and environmental conditions [52,54]. Consistent with this view, digitally mature organizations demonstrate alignment between digital strategies and core organizational objectives, supported by advanced digital infrastructure, skilled personnel, and the use of data for continuous learning and improvement [52].

In this study, digital maturity was operationalized using the HIMSS EMRAM classification. EMRAM provides a standardized, stage-based measure (stages 0-7) of digital maturity, ranging from basic EHR implementation to advanced interoperability, analytics, and patient engagement [34]. The different stages of the HIMSS EMRAM maturity model are outlined in Table 2. To further clarify the distinctions between the EMRAM stages, a matrix-style overview can be found in Multimedia Appendix 1.

**Table 2:** The maturity stages of the Electronic Medical Record Adoption Model [34].

Stage	Definition
Stage 0	The organization has not installed all of the key ancillary department systems (laboratory, pharmacy, cardiology, radiology, etc).
Stage 1	Laboratory, imaging, pharmacy, and cardiology systems produce patient-centric reports and results. Resilience management plans are in place.
Stage 2	A CDR^a^ provides access to results and reports, governance and policy controls, clinical decision support opportunities, training records, and IT security.
Stage 3	Electronic clinical documentation is accessed remotely through the CDR. Role-based access controls are in place.
Stage 4	Computerized practitioner order entry and electronic prescribing within an electronic medicine administration record. Clinical and Information governance is well defined. Monitoring of clinical outcomes and patients’ satisfaction targets.
Stage 5	Integration of data from external sources. Change in clinical parameters is continuously monitored by alerts and warnings. Telehealth and virtual care services are available. Intruder Prevention systems manage unauthorized access. Technology supports bedside processes.
Stage 6	Integration of medical devices. Health information exchange supports data sharing. Service users submit self-reported outcomes data. Wearables and implants support remote monitoring and patient management of health and care. Online services improve access and health literacy.
Stage 7	Integration of data from multiple external sources. Service users receive alerts and reminders to support self-managed care and use automated tools to measure patient outcomes. Digital infrastructure tools enable dynamic patient engagement in managing personal health and care.

^a^CDR: clinical data repository.

Conceptually, HIMSS EMRAM captures technological and informational dimensions of digital maturity, also encompassing dimensions such as infrastructure, interoperability, data analytics, and process integration, while only indirectly addressing governance and patient-centered aspects [34].

Consistent with prior EMRAM-based literature, digital maturity was operationalized dichotomously, as used in the Dutch setting before [10,12]. In line with this evidence, hospitals publicly validated at stage 6 or 7 were classified as digitally mature, and all others were categorized as lower maturity (stages 0-5). For the present study, EMRAM stages were assigned to hospitals based on publicly available press releases and announcements confirming their achievement of stage 6 or 7 certification. These announcements reflect the completion of HIMSS’s standardized assessment and external validation process. Once a hospital achieved this level, it was classified as digitally mature for that year and all subsequent years. The year of the public announcement was used as the assignment year. A table of these hospitals can be found in Multimedia Appendix 2. Most hospitals that achieved stage 6 or 7 did so before the start of the observation period (2017-2023). Consequently, their maturity status did not vary within the study window. Because the advanced EMRAM stages were reached before the performance data began, a lagged specification was not necessary.

Detailed stage-level data (stages 0-7) were not publicly available for Dutch hospitals. This absence of granular data reflects known characteristics of EMRAM self-assessment: hospitals often assess at stage 0 and mostly do not undergo reassessment until they reach level 6 [10], resulting in limited representation of stages 1 to 5 in datasets. Since EMRAM participation is voluntary and not all hospitals publicly disclose their certification activities, this approach may result in misclassification of hospitals that achieved advanced stages but did not announce them publicly.

As a robustness test, the analysis was run without hospitals that varied during the observation period to control for temporal effects.

As a further robustness test, the analysis was replicated using Newsweek’s World’s Smartest Hospitals ranking as an alternative indicator of digital maturity to assess consistency with the HIMSS EMRAM-based findings. Newsweek’s ranking is a broader composite index embedding digital innovation within overall hospital reputation, quality, and patient experience. While HIMSS EMRAM emphasizes objective system functionality and interoperability [34], Newsweek’s ranking incorporates perceptual and reputational elements [55]. Their partial conceptual overlap makes Newsweek a useful robustness check rather than a substitute for EMRAM, allowing assessment of whether findings depend on a narrowly technical versus broader digital sophistication perspective [55].

### Control Variables

Aligning with the hospital performance literature, several control variables were included: hospital size (number of beds) [12,56,57], teaching status (academic vs nonacademic) [12,42], staff-to-patient ratio [58,59], and 2 temporal controls: years (2017-2023) and a COVID-19 dummy variable (2020-2021). To account for the longitudinal character of the data and isolate the impact of the COVID-19 pandemic, this allows for distinguishing between general time trends and the exogenous shock of the COVID-19 pandemic. The COVID-19 dummy was coded as 1 for 2020-2021, corresponding to the main period of national restrictions [60]. A sensitivity analysis using 2020-2022 yielded no meaningful changes in model estimates. An operationalization table is provided in Multimedia Appendix 3 containing detailed definitions, formulas, and expected effects for all used variables.

### Analysis

A series of mixed-effects models was estimated to test the robustness of the relationship between digital maturity and hospital performance, while controlling for hospital size, teaching status, years, COVID-19, and staff-to-patient ratio, as influential factors. The unit of analysis was the hospital, with repeated annual observations spanning from 2017 to 2023. Several nested mixed-effects models were estimated to progressively include temporal and organizational controls (models 1 to 5.1). All models included random intercepts to account for between-hospital variation; year was modeled as a fixed effect, and in extended specifications, as a random effect to capture within-hospital temporal correlations.

As the more complex specification, including random slopes for time (model 5.1), did not significantly improve or alter patterns compared with the simpler model 3.1, the latter is reported for parsimony. Model 3.1 includes a random intercept for hospitals and treats year as a fixed effect, while accounting for clustering at the hospital level. The complete mixed-effects model development, as well as the fit indices (Akaike information criterion [AIC], Bayesian information criterion [BIC], and intraclass correlation coefficient [ICC]) for all outcome variables, are reported in Multimedia Appendix 4. No subgroup or interaction analyses were prespecified because the primary objective was to estimate the overall association between advanced digital maturity and hospital-level performance.

The first-order autoregressive (AR1) covariance structure was used, assuming declining correlations over longer time intervals between repeated measures. This specification balances parsimony and realistic temporal dependence compared to unstructured or compound symmetry alternatives [61]. This structure aligns with the longitudinal nature of the data as it assumes that observations closer in time (eg, in 2017 and 2018) are correlated more strongly than those further apart in time (eg, in 2017 and 2023).

Variance inflation factors (VIFs) were computed to test for multicollinearity (VIF<5) [62], and heteroskedasticity was addressed via robust specification and Bonferroni-adjusted post-hoc comparisons to adjust for multiple comparisons and control the family-wise error rate (FWER), ensuring more conservative significance testing across multiple hypothesis tests [63]. Further, a power analysis was conducted to estimate the required sample sizes for detecting incremental variance explained by digital maturity beyond the covariates by comparing a nested covariate-only model with a full model including digital maturity. Assuming α=.05 and 80% power, required sample sizes were estimated for incremental effects of Δ*R*^2^=0.01, Δ*R*^2^=0.02, and Δ*R*^2^=0.05.

To assess whether changes in HIMSS EMRAM status during the observation period influenced the findings, we conducted a sensitivity analysis excluding hospitals that were reclassified or upgraded to HIMSS stage 6 or 7 during the study period. This analysis was intended to examine the robustness of the results to potential exposure-timing effects.

Statistical significance was set at a *P* value of <.05.

All analyses were conducted using IBM SPSS Statistics for Windows (version 28.0.1.1; IBM Corp), with parameters estimated via maximum likelihood (ML). ML was selected because the analysis involved comparing multiple nested model specifications with different sets of fixed effects. ML provides valid likelihood-based comparisons (AIC, BIC, and likelihood-ratio tests) across models with differing fixed-effects structures and allows the estimation of the AR1 covariance structure used in this study [35].

The linear mixed-effects model is structured as follows:



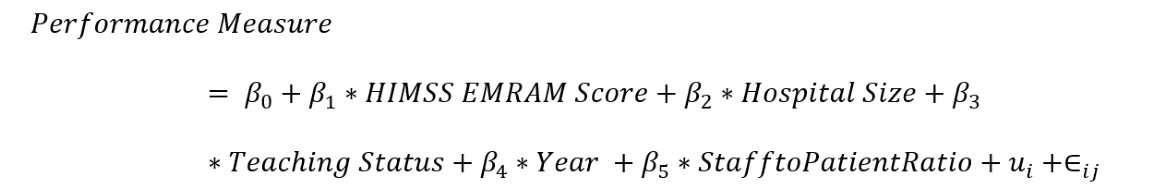



Where *β*_0_ is the intercept, *β*_1_, *β*_2_, *β*_3_, and *β*_4_ are the coefficients for the HIMSS EMRAM score and the control variables, *u_i_* represents the random intercept for the hospitals 
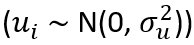
, and *ϵ* represents the error term 
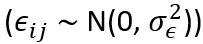
. To control the family-wise error rate arising from multiple hypothesis tests across the outcome variables, *P* values were adjusted using the Holm-Bonferroni correction procedure [63].

### Ethical Considerations

This study received ethical approval from Maastricht University (reference number FHML/HPIM/2024.600).

## Results

### Descriptive Results

Over the 7-year period (2017-2023), the number of hospitals included ranged from 66 to 74, with an average bed capacity of approximately 500 beds (Tables 3 and 4). Academic hospitals represented about 11% of the sample, remaining constant across years. The number of highly digitalized hospitals (HIMSS stage 6 or 7) ranged from 12 to 13 and remained stable.

**Table 3:** Descriptive statistics (2017-2020). The mean rate represents the percentage of academic hospitals among the total number of hospitals included. For each year, 8 academic hospitals were included.

Variables	2017, mean (SD); n	2018, mean (SD); n	2019, mean (SD); n	2020, mean (SD); n
Hospitals	0.11 (0.31); 74	0.11 (0.31); 73	0.11 (0.32); 72	0.11 (0.32); 71
Number of beds	504.08 (301.01); 74	510.90 (279.04); 73	503.03 (276.13); 72	491.06 (271.49); 71
COVID-19 year	0.00 (0.00); 74	0.00 (0.00); 73	0.00 (0.00); 72	1.00 (0.00); 71
HIMSS^a^ stage 6	1.00 (0.00); 9	1.00 (0.00); 11	1.00 (0.00); 11	1.00 (0.00); 11
HIMSS stage 7	1.00 (0.00); 1	1.00 (0.00); 1	1.00 (0.00); 1	1.00 (0.00); 2
Number of patients treated	122,787.74 (84,032.16); 74	122,968.61 (78,747.14); 70	121,005.11 (67,070.85); 70	116,357.46 (65,720.41); 67
Return on assets	0.0007 (0.19); 74	0.0234 (0.04066); 73	0.0224 (0.022); 72	0.0367 (0.159); 70
Profit margin	1.83 (2.28); 73	1.78 (2,48); 73	1.79 (1.54); 72	1.41 (1.79); 70
Asset-turnover ratio	0.64 (0.05); 73	0.07 (0.06); 72	0.033 (0.02); 72	0.1241 (0.77); 70
Personnel-expense ratio	51.28 (6.35); 73	50.29 (7.27); 72	50.64 (7.16); 69	51.68 (7.82); 70
Staff-to-patient ratio	0.0254 (0.12); 69	0.03 (0.03); 70	0.0345 (0.20); 69	0.0385 (0.021); 67
Length of stay	1.19 (1.00); 71	1.22 (1.15); 69	1.16 (1.22); 69	1.09 (0.97); 65
Absenteeism	4.75 (0.78); 73	6.89 (1.01); 73	5.27 (0.98); 72	5.76 (1.06); 71

^a^HIMSS: Healthcare Information and Management Systems Society.

**Table 4:** Descriptive statistics (2021-2023). The mean rate represents the percentage of academic hospitals among the total number of hospitals included. For each year, 8 academic hospitals were included.

Variables	2021, mean (SD); n	2022, mean (SD); n	2023, mean (SD); n
Hospitals	0.10 (0.30); 71	0.11 (0.32); 71	0.12 (0.33); 66
Number of beds	493.68 (286.20); 71	512.99 (288.95); 71	518.26 (290.24); 65
COVID-19 year	1.00 (0.00); 71	1.00 (0.00); 71	0.00 (0.00); 66
HIMSS^a^ stage 6	1.00 (0.00); 10	1.00 (0.00); 11	1.00 (0.00); 11
HIMSS stage 7	1.00 (0.00); 2	1.00 (0.00); 2	1.00 (0.00); 2
Number of patients treated	115,570.34 (66,145.21); 70	136,677.34 (73,865.06); 70	174,966.23 (184,670.24); 65
Return on assets	0.0163 (0.02); 70	0.9632 (7.94); 71	0.0196 (0.02); 66
Profit margin	1.33 (1.88); 70	2.07 (4.56); 70	2.37 (5.72); 66
Asset-turnover ratio	0.0311 (0.03); 70	0.0246 (4.56); 71	0.0242 (0.02); 66
Personnel-expense Ratio	52.18 (6.35); 70	47.02 (6.60); 69	45.96 (7.02); 66
Staff-to-patient ratio	0.0373 (0.02); 70	0.0190 (0.03); 70	0.0144 (0.01); 65
Length of stay	1.09 (1.06); 67	–^b^	–
Absenteeism	6.22 (1.21); 71	7.30 (1.36); 71	6.24 (1.44); 66

^a^HIMSS: Healthcare Information and Management Systems Society.

^b^Not applicable.

Multimedia Appendix 5 shows the descriptive characteristics of hospitals stratified by digital maturity status. Hospitals with EMRAM stage 6 or 7 were, on average, larger than hospitals at lower stages, with a higher number of beds and employees, and treated substantially more patients annually. Digitally mature hospitals were also more frequently teaching hospitals.

Financial indicators such as profit margin and ROA fluctuated moderately over time, with declines during the COVID-19 period (2020-2021) and recovery thereafter. The descriptive statistics for ROA in 2022 were influenced by a small number of extreme observations caused by unusually small asset denominators. However, the distribution remained centered near zero (median 0.013; range −0.05 to 0.020), and these values did not materially affect the regression results. Operational measures, including the number of patients treated and personnel-expense ratio, also varied in line with pandemic-related disruptions.

### Multilevel Model Results

Across the 7-year period (2017-2023), the results do not support hypothesis 1, which predicted superior financial and operational performance among hospitals with higher digital maturity. Specifically, higher digital maturity (HIMSS EMRAM stage 6 or 7) was not associated with improvements in profit margins, ROA, or asset turnover compared with less mature hospitals (Table 5). Hospitals with higher digital maturity initially appeared to treat more patients (*β*=31,664.318; 95% CI 8392.095 to 54,936.540; *P*=.008). However, this effect lost significance after Holm-Bonferroni correction for multiple testing (adjusted *P*=.44). No associations were observed with length of stay (Table 6), indicating that advanced EHR integration did not measurably increase patient throughput. This suggests that digital maturity might primarily impact the outpatient setting. These findings indicate that advanced digital maturity was not associated with higher system-wide patient throughput or reduced inpatient usage, providing no empirical support for the operational efficiency component of hypothesis 1. Furthermore, no significant relationships were found between digital maturity and absenteeism (hypothesis 2; Table 6), implying that workforce well-being and efficiency were unaffected by higher EMRAM stages.

**Table 5:** Results of the full separate univariate mixed models predicting the effect of a high degree of digitalization on the hospital performance measures with time as a fixed effect. Variance inflation factor values are reported in Multimedia Appendix 6. All variance inflation factor values were below the threshold of 5 (mean 1.8), indicating no relevant multicollinearity.

Parameter		Profit margin	Return on assets	Personnel-expense ratio	Asset-turnover ratio
Intercept, *β* (95% CI)		15.427 (−148.167 to 179.021)	2.106 (−0.519 to 4.731)	546.383 (143.895 to 948.870)^a^	8.418 (5.884 to 10.951)^b^
**Control variables, *β* (95% CI)**					
	Academic hospital	−0.209 (−0.898 to 0.479)	−0.007 (−0.019 to 0.004)	3.583 (0.903 to 6.263)^a^	−0.009 (−0.018 to 0.000)
	Size (number of beds)	0.000 (−0.001 to 0.002)	0.000 (−0.000 to 0.000)	−0.001 (−0.004 to 0.002)	0.000 (−0.000 to 0.000)
	Staff-to-patient ratio	0.581 (−7.492 to 8.654)	0.172 (0.053 to 0.290)^a^	−0.054 (−10.806 to 10.698)	0.006 (−0.054 to 0.066)
**Independent variables, *β* (95% CI)**					
	Year	−0.007 (−0.088 to 0.074)	−0.001 (−0.002 to 0.000)	−0.246 (−0.445 to −0.047)^c^	−0.004 (−0.005 to −0.003)^b^
	COVID-19 year	−0.539 (−0.850 to −0.229)^b^	−0.004 (−0.009 to −0.000)^c^	1.866 (1.313 to 2.418)^b^	−0.001 (−0.005 to 0.004)
	Dummy high degree of digitalization	−0.576 (−2.268 to 1.116)	−0.002 (−0.011 to 0.008)	0.808 (−1.385 to 3.000)	0.001 (−0.007 to 0.009)
ICC^d^		0.38	0.00	0.50	0.00
AIC^e^		2106.80	−1293.92	2495.05	−1970.09
BIC^f^		2139.90	−1260.80	2528.08	−1937.29

^a^*P*<.01.

^b^*P*<.001.

^c^*P*<.05.

^d^ICC: intraclass correlation coefficient.

^e^AIC: Akaike information criterion.

^f^BIC: Bayesian information criterion.

**Table 6:** Results of the full separate univariate mixed models predicting the effect of a high degree of digitalization on the hospital performance measures with time as a fixed effect. Variance inflation factor values are reported in Multimedia Appendix 6. All variance inflation factor values were below the threshold of 5 (mean 1.8), indicating no relevant multicollinearity.

Parameter		Number of patients treated	Length of stay	Absenteeism
Intercept, *β* (95% CI)		91,594.179 (76,164.253 to 107,027.104)^a^	98.410 (24.469 to 172,351)^b^	−730.443 (−818.561 to −642.325)^a^
**Control variables, *β* (95% CI)**				
	Academic hospital	−2622.911 (−16,442.066 to 11,196.243)	−0.146 (−0.521 to 0.229)	−0.233 (−0.722 to 0.275)
	Size (number of beds)	86.749 (66.622 to 106.877)^a^	−0.000 (0.000 to 0.000)	0.000 (−0.001 to −0.001)
	Staff-to-patient ratio	−473,771.873 (−617,885.714 to −329,658.031)^a^	18.331 (16.678 to 19.984)^a^	−0.274 (−3.130 to 2.582)
**Independent variables, *β* (95% CI)**				
	Year	0.003	−0.048 (−0.085 to −0.012)^c^	0.321 (0.266 to 0.365)^a^
	COVID-19 year	−4359.680 (−6727.588 to −1991.772)^a^	−0.101 (−0.197 to −0.006)^c^	−0.075 (−0.259 to 0.110)
	Dummy high degree of digitalization	31,664.318 (8392.095 to 54,936.540)^b^	−0.252 (−0.529 to 0.023)	−0.159 (−0.585 to 0.266)
ICC^d^		0.83	0.81	0.10
AIC^e^		11,129.76	246.97	1293.75
BIC^f^		11,162.91	269.75	1326.88

^a^*P*<.001.

^b^*P*<.01.

^c^*P*<.05.

^d^ICC: intraclass correlation coefficient.

^e^AIC: Akaike information criterion.

^f^BIC: Bayesian information criterion.

### Model Diagnostics and Robustness Checks

Model diagnostics indicated that the regression assumptions were adequately met. VIFs for all predictors were below 5 (mean 1.8), suggesting no multicollinearity (Multimedia Appendix 6). Residual inspection showed approximate normality and homoscedasticity, supporting model adequacy. Model fit indices (AIC and BIC) favored the fully adjusted models, including year and staff-to-patient ratio covariates.

After applying the Holm-Bonferroni correction for multiple comparisons (Table 7), none of the associations between high digital maturity and hospital performance remained statistically significant, including the initially positive effect on the number of patients treated (adjusted *P*=.44). Replication using Newsweek’s World’s Smartest Hospitals ranking as an alternative proxy for digital maturity yielded consistent null results, confirming the robustness of the findings (Multimedia Appendix 7).

**Table 7:** Holm-Bonferroni correction for the degree of digitalization with year as fixed effect.

HIMSS^a^ degree of digitalization	Raw *P* value	Corrected *P* value	Significance
Profit margin	.50	>.99	No
Return on assets	.73	>.99	No
Personnel-expense ratio	.47	>.99	No
Asset-turnover ratio	.80	>.99	No
Number of patients treated	.008	.44	No
Length of stay	.07	>.99	No
Absenteeism	.46	>.99	No

^a^HIMSS: Healthcare Information and Management Systems Society.

The power analysis indicated that 1455 hospital-year observations would be required to detect a very small incremental effect of digital maturity (Δ*R*^2^=0.01) and 286 observations to detect a moderate incremental effect (Δ*R*^2^=0.05; Multimedia Appendix 8).

The sensitivity analysis excluding hospitals that were reclassified or upgraded to HIMSS stage 6 or 7 during the study period yielded results consistent with the main analysis. This suggests that the main findings were not driven by hospitals whose digital maturity classification changed during the observation period (Multimedia Appendix 9).

## Discussion

### Digital Maturity and the Productivity Paradox

Taking a longitudinal approach, looking at Dutch hospital performance over a 7-year period, we examined whether high digital maturity, operationalized as achieving HIMSS EMRAM stage 6 or 7, was associated with improved profit margins, ROA, asset-turnover ratio, personnel-expense ratio, length of stay, or absenteeism. In fully adjusted mixed-effects models, we did not detect statistically significant associations between high digital maturity and the hospital performance measures. Digitally mature hospitals initially appeared to treat a higher number of patients per year. However, this association did not remain statistically significant after applying correction. These findings suggest that, within the Dutch context over the study period, we did not find robust evidence that achieving higher EMRAM stages was associated with substantial improvements in the aggregate performance indicators examined in this study.

The findings can be interpreted through 3 complementary explanations. First, they are consistent with the IT productivity paradox and the possibility that digital benefits are delayed, offset, or not captured by aggregate performance indicators. Second, they may reflect the distinction between technical maturity and the organizational transformation required to generate value from digital systems. Third, they should be interpreted in light of the Dutch high-baseline digitalization context, where limited contrast between maturity groups may make incremental performance differences difficult to detect.

### Productivity Paradox and Diluted Performance Effects

First, these findings are best interpreted through the lens of the IT productivity paradox, in which substantial technology investments fail to produce comparative performance improvements at the organizational level [20-23,64]. This aligns with earlier systematic reviews, which found that while EHR adoption is frequently associated with improvements in documentation and safety, evidence for broad financial or operational gains remains limited and inconsistent [65,66]. One explanation proposed in prior work is that digital systems may initially increase the administrative workload and disrupt established workflows [7,33,67,68]. This may lead to temporary productivity loss before potential efficiency gains materialize [19,64]. Benefits may also arise in domains not captured by traditional performance indicators, such as patient safety, care coordination, or even financial and operational measures [10,69]. Therefore, the absence of statistically robust associations between advanced EMRAM stages and the examined hospital performance indicators should not be interpreted as definitive evidence that digital maturity has no impact on hospital performance. Rather, it suggests that benefits may be delayed or offset by new forms of organizational burden, such as increased documentation requirements, workflow complexity, or cognitive load [19]. At the hospital level, efficiency gains in some areas may be counterbalanced by rising costs or constraints elsewhere, resulting in neutral net effects when using conventional performance indicators.

Relatedly, building on the productivity paradox, these findings suggest that performance impacts of advanced digital maturity are often selective or context-specific and difficult to detect using aggregated hospital-level indicators. For example, van Poelgeest et al [12] found shorter postoperative stays in Dutch hospitals with higher EMRAM scores. In contrast, our study did not observe reductions in length of stay. This suggests that the value of advanced digital maturity may be more visible in specific clinical or experiential domains than in hospital-wide performance metrics. Such dilution effects are particularly plausible in health care systems like the Netherlands, where baseline EHR adoption is already high. This aligns with prior research showing that EMRAM-related performance effects often manifest in specific clinical or safety domains rather than at the hospital level [10,12,13]. The performance indicators used in this study may not fully capture where digital value is created in mature digital environments.

### Technical Maturity Without Operational Transformation

Second, EMRAM primarily captures technical maturity [50,53], but digitalization also depends on strategic alignment, organizational processes, and human capabilities. Consequently, hospitals classified as “digitally mature” may differ substantially in the extent to which digital systems are embedded into clinical and administrative workflows, supported by process redesign, and aligned with strategic objectives, a variation that EMRAM is not designed to capture [32,50,70].

For example, Srivastava et al [71] found that leadership engagement, system configuration, and user familiarity were stronger predictors of satisfaction and efficiency than EMRAM stage alone. Thus, technological maturity appears to be a necessary but insufficient condition for achieving digital value. Without parallel development of organizational capabilities, workforce readiness, and process redesign, hospitals risk stagnating in a state of “digital inertia,” in which substantial investments in digital infrastructure fail to translate into observable performance improvements. This is consistent with the sociotechnical systems theory, in which the effectiveness of digital investments depends on aligning technological tools with human, cultural, and organizational subsystems that enable their productive use [72].

Furthermore, comparative evidence highlights that the relationship between digitalization and performance is mediated by organizational and financial context. A study of German hospitals showed that organizations with greater profitability and network affiliations achieved higher digital maturity and better leveraged centralized IT governance [14]. Conversely, smaller or resource-limited hospitals may lack the absorptive capacity to translate digital investments into measurable outcomes [71,73]. In our sample, hospitals with advanced digital maturity (EMRAM stage 6 or 7) were structurally different from lower-stage hospitals. They were larger, more frequently classified as teaching hospitals, and treated higher patient volumes (Multimedia Appendix 5). However, these structural differences did not translate into superior financial, operational, or workforce performance outcomes.

### Limited Contrast in High-Baseline Contexts

Third, research has shown that associations between advanced EMRAM stages and performance outcomes are highly stage dependent. For example, Jarvis et al [13] and Snowdon et al [10] reported positive associations between advanced EMRAM stages and process-of-care outcomes or safety outcomes, but the effects were strongest only for stage 7 hospitals. Notably, hospitals at stage 6 did not significantly differ from lower-stage hospitals in these studies [10,13]. Our findings are consistent with the notion that measurable performance benefits may emerge only when digital integration is fully optimized.

At the same time, this pattern highlights a broader methodological concern in EMRAM literature: what constitutes a meaningful comparison of maturity stages. Comparisons between stage 7 hospitals and those at much lower stages (eg, stages 1 and 2) provide analytically striking results [10] but reflect contrasts that are increasingly rare in health systems characterized by widespread EHR adoption. In highly digitalized contexts, such as the Netherlands [2], hospitals are more likely to cluster at intermediate or advanced levels of digital maturity. This study, therefore, provides a more realistic benchmark for assessing the incremental value of advanced digital maturity in a high-baseline setting, focusing on marginal gains beyond widespread EHR adoption rather than on adoption-phase effects.

This study contributes to the digital health literature by extending EMRAM-based research beyond the adoption phase and into a postadoption, high-maturity context. By demonstrating that higher EMRAM stages were not robustly associated with improvements in aggregated financial, operational, or workforce outcomes, this study provides empirical support for the productivity paradox in hospital digitalization. This underscores that, in mature digital environments, further gains from technical advancement alone are likely to be limited and dependent on a broader sociotechnical transformation.

### Implications: From Adoption to Value Creation

The absence of performance differences between highly digitally mature hospitals, which were publicly validated, and their less mature peers suggests that Dutch hospitals may have reached a stage where the technical infrastructure is largely in place, but where additional gains depend on complementary organizational and strategic capabilities. This supports the interpretation that hospitals are operating in a “postadoption” phase in which further investments in core digital infrastructure yield diminishing marginal returns unless accompanied by changes in governance, workflow design, and workforce capabilities.

First, from a policy perspective, national digital health strategies should move beyond incentivizing system implementation toward supporting the organizational conditions required for value realization. This implies that policy efforts should include investments and incentives to strengthen workforce capabilities and support process redesign, thereby enabling hospitals to translate advanced digital maturity into measurable performance gains.

Second, the findings underscore the relevance of the IT productivity paradox in the current hospital setting. It appears that technological maturity is a necessary but insufficient condition for productivity gains. Without parallel investments in clinical engagement, digital skills, and workflow integration, hospitals risk accumulating technical capacity without realizing its potential benefits. For hospital management, this implies that digital transformation initiatives should be evaluated not only on technical milestones (eg, EMRAM stages) but also on how effectively digital tools are embedded in day-to-day clinical and administrative processes.

Third, the results highlight the need for realistic expectations for measurable performance gains: in highly digitalized systems, additional gains from incremental increases in technical maturity may be subtle or domain-specific rather than visible in hospital-level financial, operational, or workforce indicators. This has implications for how digital success is measured and communicated. Overreliance on aggregated performance indicators may obscure where digital value materializes, reinforcing the need for more comprehensive evaluation frameworks.

Fourth, the implications of this study extend to future innovations, for example, learning health systems, advanced analytics, and artificial intelligence. While a robust digital infrastructure is a prerequisite for these developments, our findings suggest that infrastructure alone will not guarantee performance improvements. Emerging technologies may amplify existing organizational shortcomings if issues of governance, workforce readiness, and process alignment are not addressed. Policymakers and hospital leaders should therefore view digital maturity not as an endpoint, but as an enabling condition that must be actively leveraged through organizational transformation to achieve sustainable value creation.

### Limitations

This study is subject to several limitations. First, EMRAM data were operationalized dichotomously, comparing hospitals with publicly documented stage 6 or 7 validation with hospitals at EMRAM stage ≤5 or without public evidence of advanced validation. This approach reduces granularity and may obscure relevant differences between adjacent maturity stages. This is particularly important in the Dutch context, where baseline EHR adoption is high, and hospitals without stage 6 or 7 validation may nevertheless have substantial digital infrastructure. Consequently, the comparison may reflect marginal differences between already relatively digitalized hospitals rather than a contrast between digitally mature and digitally immature organizations. This limited exposure contrast may have contributed to a ceiling effect and reduced the ability to detect small incremental performance differences. In addition, public EMRAM validation dates may not coincide exactly with the actual timing of technological deployment or organizational implementation. This may introduce timing misclassification, particularly if digital capabilities were implemented before public validation or if performance effects emerged only after a longer adaptation period. However, this limitation is partly mitigated by the fact that almost all hospitals classified as stage 6 or 7 had achieved validation before the start of the observation period. Residual temporal uncertainty, therefore, remains possible, but it is unlikely to fully explain the observed absence of robust associations.

Second, EMRAM primarily captures technical and interoperability capabilities and provides limited insight into organizational, cultural, or workforce-related dimensions of digital transformation. This is important because the relationship between digital maturity and hospital performance may be mediated by organizational mechanisms such as implementation quality, workflow redesign, interoperability in daily practice, staff adoption, data-use capabilities, leadership engagement, and strategic alignment. These constructs were not directly measured in the available annual-report data. Therefore, formal mediation analyses were not conducted because the dataset did not provide valid intermediate variables with a clear exposure-mediator-outcome sequence. Variables such as length of stay and number of patients treated could theoretically be part of a broader value-creation pathway, but they were specified as primary performance outcomes in this study and were therefore not suitable for use as mediators in the same analytical framework. As a result, the analysis may underestimate the broader socio-technical conditions required for digital value creation.

Third, only 13 hospitals achieved EMRAM stage 6 or 7. Although the longitudinal design increases statistical observations, the effective statistical power was constrained by the small number of hospitals in the advanced digital-maturity group, the imbalance between exposure groups, and the clustered structure of repeated observations within hospitals. Consequently, the study was better positioned to detect moderate-to-large associations than small incremental effects. The null findings reported here should therefore be interpreted as an absence of statistically detectable associations rather than evidence of equivalence.

Fourth, while the models included key structural controls, residual confounding cannot be ruled out. Factors such as case-mix complexity, network participation, managerial practices, and regional competition were not available in the dataset and may influence both digital investment and performance. Finally, missing data were handled via available-case analysis without imputation. Although missingness was relatively limited, any systematic pattern in omitted observations could introduce bias.

### Future Research: Moving Beyond the Paradox

Future research should extend this study by testing nonlinear or inverse (U-shaped) relationships between digital maturity and hospital performance. Such analyses could reveal whether performance gains diminish or even reverse at higher EMRAM stages, investigating the possibility of a maturity plateau. Moreover, the productivity paradox should be addressed more directly by linking digital maturity to an interaction mix of quality, safety, and patient experience indicators, rather than financial and operational metrics alone.

Access to granular HIMSS EMRAM data would allow modeling maturity as a categorical variable (stages 0-7), capturing stage-specific and lagged effects that may appear only after a temporal delay. To broaden the conceptual scope, future research should integrate more holistic maturity frameworks (eg, Mettler and Pinto [53]; National Health Service [NHS] Digital Maturity Index) encompassing leadership, workforce readiness, and change management.

Future studies with access to valid organizational and process-level measures should also conduct mediation analyses to examine whether digital maturity affects hospital performance indirectly through implementation quality, workflow redesign, interoperability in daily practice, staff adoption, data-use capabilities, leadership engagement, and strategic alignment. Finally, mixed methods studies combining quantitative performance data with qualitative insights into leadership, digital literacy, and process redesign are needed to uncover the mechanisms through which digital maturity translates or fails to translate into organizational value.

### Conclusion

In this longitudinal study of Dutch hospitals spanning over 7 years, no statistically robust associations between advanced digital maturity (EMRAM stage 6 or 7) and hospital-level financial, operational, or workforce performance were found. While digitally mature hospitals initially appeared to treat more patients, this pattern did not remain significant after adjustment for multiple testing, and all other performance indicators showed no significant differences. These results suggest that, in a highly digitalized health system, EMRAM status alone may not capture the organizational factors necessary to translate digital capability into measurable performance gains. Future work using more granular maturity measures, quality and safety outcomes, and mixed methods designs is needed to clarify when and how digital maturity produces value for hospitals and patients.

## Appendix

Multimedia Appendix 1 Matrix-style overview of the Healthcare Information and Management Systems Society (HIMSS) Electronic Medical Record Adoption Model (EMRAM) stages.

Multimedia Appendix 2 Dutch Hospitals with Healthcare Information and Management Systems Society (HIMSS) Electronic Medical Record Adoption Model (EMRAM) stages 6 or 7.

Multimedia Appendix 3 Extended variable operationalization table.

Multimedia Appendix 4 Development of the mixed effect models.

Multimedia Appendix 5 Comparison of hospital characteristics by Healthcare Information and Management Systems Society (HIMSS) Electronic Medical Record Adoption Model (EMRAM) stage: stages ≤5 vs stages 6 and 7.

Multimedia Appendix 6 Variance inflation factor analysis.

Multimedia Appendix 7 Model development sensitivity analysis Newsweeks World Smartest Hospital Ranking.

Multimedia Appendix 8 Results of the power analysis.

Multimedia Appendix 9 Development of mixed-effect models without changes in during the observation period.

## Abbreviations

AIC: Akaike information criterion
AR1: first-order autoregressive
BIC: Bayesian information criterion
CIBG: Dutch National Annual Healthcare Reports Database
DRG: diagnosis-related group
EHR: electronic health record
EMRAM: Electronic Medical Record Adoption Model
FWER: family-wise error rate
HIMSS: Healthcare Information and Management Systems Society
ICC: intraclass correlation coefficient
ML: maximum likelihood
NHS: National Health Service
ROA: return on assets
VIF: variance inflation factor

## Data Availability

The datasets analyzed during the current study are publicly available from the Dutch National Annual Healthcare Reports Database (CIBG) [74]. Derived datasets generated and SPSS analysis syntax during the study are available from the corresponding author upon reasonable request.
