# Supplementary material for: Impact of Digitalization on Performance of Dutch Hospitals: Longitudinal Quantitative Study
Source: J Med Internet Res. 2026 Sep 29;28:e94896. doi: 10.2196/94896 (PMC13662437; doi:10.2196/94896)
Supplement: Supplementary file 1 [file jmir_v28i1e94896_app1.docx]

| **The Maturity Stages of the Electronic Medical Records Adoption Model** (Own representation) **[1]**  – = absent or not expected; ○ = emerging or partially implemented; ● = implemented; ▲ = advanced, integrated, or continuously used. | | | | | | | | |
| --- | --- | --- | --- | --- | --- | --- | --- | --- |
| **Capability domain** | **Stage 0** | **Stage 1** | **Stage 2** | **Stage 3** | **Stage 4** | **Stage 5** | **Stage 6** | **Stage 7** |
| **Ancillary departmental Systems** | **-** | **●** | **●** | **●** | **●** | **●** | **●** | **●** |
| **Clinical data repository** | **-** | **○** | **●** | **●** | **●** | **●** | **●** | **●** |
| **Electronic clinical documentation** | **-** | **-** | **○** | **●** | **●** | **●** | **●** | **●** |
| **CPOE and e-prescribing** | **-** | **-** | **-** | **○** | **●** | **●** | **●** | **●** |
| **External data integration** | **-** | **-** | **-** | **-** | **○** | **●** | **●** | **▲** |
| **Medical device integration** | **-** | **-** | **-** | **-** | **-** | **○** | **●** | **▲** |
| **Patient-generated health data** | **-** | **-** | **-** | **-** | **-** | **○** | **●** | **▲** |
| **Dynamic patient engagement** | **-** | **-** | **-** | **-** | **-** | **○** | **○** | **▲** |

1.	*HIMSS*. Electronic Medical Record Adoption Model (EMRAM). 2024.
