# Supplementary material for: Impact of Digitalization on Performance of Dutch Hospitals: Longitudinal Quantitative Study
Source: J Med Internet Res. 2026 Sep 29;28:e94896. doi: 10.2196/94896 (PMC13662437; doi:10.2196/94896)
Supplement: Supplementary file 2 [file jmir_v28i1e94896_app2.docx]

| **Hospitals with HIMSS Stages 6 or 7 in the Netherlands** | | | | |
| --- | --- | --- | --- | --- |
| **Name** | **First public Stage 6 / year** | **Upgrade / Reclassification year (if applicable)** | **Exposure Status at baseline** | **Source** |
| **Spaarne Ziekenhuis / Spaare Gasthuis** | 2011 | - | Advanced before baseline | [Spaarne Ziekenhuis ontvangt onderscheiding van HIMSS Analytics Europe voor inzet EPD - MedicalFacts.nl](https://www.medicalfacts.nl/2011/07/08/spaarne-ziekenhuis-ontvangt-onderscheiding-van-himss-analytics-europe-voor-inzet-epd/) |
| **Westfiesgasthuis (Hoorn) ^1^** | 2012 | - | Advanced before baseline | [Westfriesgasthuis behaalt prestigieuze onderscheiding voor verregaande automatisering patiÃ«ntendossiers - MedicalFacts.nl](https://www.medicalfacts.nl/2012/10/12/westfriesgasthuis-behaalt-prestigieuze-onderscheiding-voor-verregaande-automatisering-patientendossiers/) |
| **Sint Lucas Andreas Ziekenhuis^2^** | 2012 | 2018 | Change within the baseline | [Sint Lucas Andreas Ziekenhuis krijgt EPD-award - Computable.nl](https://www.computable.nl/2012/11/14/sint-lucas-andreas-ziekenhuis-krijgt-epd-award/)  [HIMSS22 Europe Celebrates Healthcare Systems Validated at EMRAM Stages 6 and 7 \| HIMSS](https://iowa.himss.org/news/himss22-europe-celebrates-healthcare-systems-validated-emram-stages-6-and-7) |
| **Amphia Ziekenhuis** | 2013 | - | Advanced before baseline | [Amphia bereikt HIMSS stage 6 met elektronisch patiëntendossier (EPD) - MedicalFacts.nl](https://www.medicalfacts.nl/2013/11/14/198229/) |
| **Leiden University Medical Center** | 2013 | - | Advanced before baseline | [LUMC krijgt eveneens prestigieus ICT-certificaat - Skipr](https://www.skipr.nl/nieuws/lumc-krijgt-eveneens-prestigieus-ict-certificaat/) |
| **University Medical Center Utrecht** | 2013 | - | Advanced before baseline | [UMC Utrecht verdient prestigieus ICT-certificaat - Skipr](https://www.skipr.nl/nieuws/umc-utrecht-verdient-prestigieus-ict-certificaat/) |
| **Ziekenhuisgroep Twente – ZGT** | 2014 | - | Advanced before baseline | [Hoge onderscheiding voor ZGT - MedicalFacts.nl](https://www.medicalfacts.nl/2014/03/18/hoge-onderscheiding-voor-zgt/) |
| **Maasstad Ziekenhuis** | 2014 | - | Advanced before baseline | [Maasstad Ziekenhuis ontvangt award voor elektronisch patiëntendossier - MedicalFacts.nl](https://www.medicalfacts.nl/2014/04/03/maasstad-ziekenhuis-ontvangt-award-voor-elektronisch-patientendossier/) |
| **Zaans Medisch Centrum** | 2014 | - | Advanced before baseline | [epd Archieven - Page 39 of 126 - Zorgvisie](https://www.zorgvisie.nl/tag/epd/page/39/) |
| **Jeroen Bosch Ziekenhuis** | 2015 | - | Advanced before baseline | ['Bij het EPD is de balans tussen privacy en medische efficiëntie doorgeschoten' - IT Pro - Nieuws - Tweakers](https://tweakers.net/nieuws/121403/bij-het-epd-is-de-balans-tussen-privacy-en-medische-efficientie-doorgeschoten.html) |
| **Radboud University Medical Center** | 2015 | 2017 (Stage 7) | Change within baseline | [Radboud University Medical Center Becomes First Hospital in the Netherlands to Win HIMSS Stage 7 Award](https://www.digitalhealthnews.eu/industry/4253-radboud-university-medical-center-becomes-first-hospital-in-the-netherlands-to-win-himss-stage-7-award)  [2017 EMRAM trailblazers \| Healthcare IT News](https://www.healthcareitnews.com/news/2017-emram-trailblazers) |
| **Stichting Antonius Ziekenhuis** | 2016 | - | Advanced before baseline | [HIMSS EMRAM Stage 6 & 7 Community \| HIMSS Europe](https://web.archive.org/web/20161210030819/http:/www.himss.eu/communities/himss-emram-stage-6-7-community) |
| **St. Jansdal Ziekenhuis** | 2016 | 2017 (Stage 7) | Change within the baseline | [‘Opereer agile en excelleer aan de zachte kant’ \| ICT&health](https://icthealth.nl/magazine/editie-02-2017/opereer-agile-en-excelleer-aan-de-zachte-kant) |

^1^ Merged to Dijklander Ziekenhuis

^2^ Merged to OLVG
