# Supplementary material for: Impact of Digitalization on Performance of Dutch Hospitals: Longitudinal Quantitative Study
Source: J Med Internet Res. 2026 Sep 29;28:e94896. doi: 10.2196/94896 (PMC13662437; doi:10.2196/94896)
Supplement: Supplementary file 3 [file jmir_v28i1e94896_app3.docx]

**Table B1. Operationalization of Variables**

| **Variable** | **Type** | **Definition / Formula** | **Source** | **Expected Effect** |
| --- | --- | --- | --- | --- |
| Profit Marign | Dependent (Financial Performance) | Ratio of net income to total revenue | [1-3] | ↑ Higher digital maturity is expected to improve financial efficiency |
| Return on Assets (ROA) | Dependent (Financial Performance) | Results after taxes / Total assets | [4-6] | ↑ Improved asset utilization through digital integration |
| Personnel-Expense-Ratio | Dependent (Cost Efficiency) | (Personnel costs / Operating revenue) x 100 | [4] | ↓ Expected reduction through workflow efficiency |
| Asset-Turnover-Ratio | Dependent (Operational Efficiency) | Total revenue / Total assets | [4, 7, 8] | ↑ Increased asset productivity via digitalized processes |
| Number of Patients Treated | Dependent (Service Delivery Efficiency | Total annual inpatient and outpatient cases treated | [4, 9, 10] | ↑ Higher throughput because of streamlined digital workflows |
| Length of Stay | Dependent (Operational Efficiency) | Total inpatient days / Number of discharges | [4, 9, 11-13] | ↓ Expected shorter stays through process optimization |
| Absenteeism Rate | Dependent (Workforce Outcome) | Percentage of total staff absent (sick leave) per year | [4, 14, 15] | ↑ Improved working conditions due to digital support tools. |
| Digital Maturity (HIMSS EMRAM Score) | Independent Variable | Dichotomous variable:  0 = EMRAM Stage 0–5,  1 = EMRAM Stage 6–7 | [16, 17] | ↑ Positive relationship with efficiency and performance outcomes |
| Hospital Size | Control Variable | Total number of hospital beds | [4, 9, 18, 19] | ↑ Larger hospitals may perform better due to economies of scale |
| Teaching Status | Control Variable | Dummy:  0 = non-teaching hospital,  1 = academic hospital | [4, 8, 9] | - teaching status may face higher costs, but higher quality |
| Staff-to-Patient-Ratio | Control Variable | Total FTE hospital staff / Total patients treated | [4, 20, 21] | ↑ High ratio could confound efficiency measures |
| Year | Control Variable (Time Trend) | Continuous variable (2017-2023) | Study design | - Captures temporal effects |
| Covid-19 Year | Control Variable (Exogenous Shock) | Dummy:  1 = 2020–2021,  0 = otherwise | Study design; [6, 22] | ↓ Negative effect expected due to pandemic disruptions |

**Table B2. General data preparation and sample construct**

| **General data preparation and sample construct** | | |
| --- | --- | --- |
| **Step** | **Source field items** | **Coding / Cleaning rule** |
| Insitutional Identifier | 2017–2019: *c_kvk*; 2020–2023: *ExternalOrganizationId* | Used as the institutional identifier for harmonizing hospital records across years. Manual identifier corrections were applied where needed, for example Tergooi in 2020 and Radboud UMC in 2021. |
| Hospital name | 2017–2019: *c_naam*; 2020–2023: *Name* | Used for manual verification of institutional identity, exclusion of non-hospital entities, and correction of organizational identifiers where needed. |
| Hospital eligibility 2017 – 2018 | *Q_TypeWTZi_Ans_TypeWTZi#0; Q_TypeWTZi_Ans_TypeWTZi#2* | Records were retained if *Q_TypeWTZi_Ans_TypeWTZi#0* = "*ja*" or *Q_TypeWTZi_Ans_TypeWTZi#2* = "*ja*". |
| Hospital eligibility 2019 | *Q_TypeWTZi_Ans_TypeWTZi_0; Q_TypeWTZi_Ans_TypeWTZi_2* | Records were retained if the hospital or UMC indicator equalled 1. |
| Hospital eligibility 2020 | *Q_TypeWTZi_MSI_0; Q_TypeWTZi_MSI_2* | Records were retained if *Q_TypeWTZi_MSI_0 = 1* or *Q_TypeWTZi_MSI_2 = 1*. |
| Hospital eligibility 2021 | *entMain_Typering_ent_Typering_0; entMain_Typering_ent_Typering_2* | Variables were recoded into *UMC* and *Hospital*. Records were retained if *UMC = 1* or *Hospital = 1*. Named non-hospital organizations were removed, and only records with valid numeric organizational identifiers were retained. |
| Hospital eligibility 2022 - 2023 | *ExternalOrganizationId* | Records were retained if the *ExternalOrganizationId* matched the predefined list of eligible hospital organizations. |
| Manual Corrections | Selected *c_kvk* / *ExternalOrganizationId* and *Name* values | Manual corrections were applied to selected hospital records where source values were missing or inconsistent. |
| Distribution and plausibility checks | Derived analytical variables | Descriptive statistics and histograms were inspected after variable derivation to identify implausible values, missingness, and distributional issues. |
| Missing Values | All variables | Variables were set to missing where required source fields were unavailable. For some ratio variables, denominators equal to zero were explicitly set to system missing. Models used available-case analysis. |

**Table B3. Variable derivation**

| **Variable derivation** | | | |
| --- | --- | --- | --- |
| **Variable** | **Source field item(s)** | **Formula / Coding rule** | **Cleaning/harmonization rule** |
| Profit Margin | 2017: *jeu11500_jeu11501, jeu10800_jeu10800; 2018–2021: jeu11520_jeu11520* or *jeu10800_jeu10800;* 2022–2023: *qResultaatNaBelastingen_0, qSomBedrijfsopbrengsten_0* | *Profit_margin = Result_after_taxes / Sum_Operating_revenues × 100* | Derived only when the results after taxes and operating revenues were available. Where explicitly coded, observations with *Sum_Operating_revenues* *= 0* were set to system missing. |
| Return on Assets | 2017–2021: *jeu11500_jeu11501 or jeu11520_jeu11520, jeu09300_jeu09300*; 2022–2023: *qResultaatNaBelastingen_0, qTotaalActiva_0* | *ROA = Result_after_taxes / Total_assets* | Derived only when the result after taxes and total assets were available. Records with missing or implausible denominator values were inspected using descriptives and histograms. |
| Personnel-expense- ratio | 2017: *jeu13251_jeu13251, jeu10800_jeu10800;* 2018: *jeu10900_jeu10900, jeu10800_jeu10800;* 2020–2021: *jeu13251_jeu13251, jeu10800_jeu10800;* 2022–2023: *qBedrijfslastLonen_0, qBedrijfslastSociaal_0, qBedrijfslastPensioen_0, qSomBedrijfsopbrengsten_0* | 2017/2020/2021: *Total_Personnel_Expenses / Sum_Operating_revenues × 100*; 2018: *PersonnelCosts / Sum_Operating_revenues × 100;* 2022–2023: *(qBedrijfslastLonen_0 + qBedrijfslastSociaal_0 + qBedrijfslastPensioen_0) / qSomBedrijfsopbrengsten_0 × 100* | Personnel-cost fields changed over time. Year-specific fields were harmonized into total personnel costs before calculating the ratio. Derived only when personnel costs and operating revenues were available. |
| Asset-turnover-ratio | 2017–2021: jeu10800_jeu10800 or jeu11210_jeu11210, jeu09300_jeu09300; 2022–2023: qBedrijfsresultaat_0 or qSomBedrijfsopbrengsten_0, qTotaalActiva_0  Year-specific CIBG operating-revenue and total-assets fields, harmonized as *Total_operating_revenue / Sum_Operating_revenues and Total_assets* | *Asset-turnover ratio = total operating revenue / total assets* | Source field names differed across years and were harmonized to the same analytical construct before calculation. Derived only when both the revenue and total-assets fields were available. |
| Number of patients treated | 2017: *qap10510_qap10510*; 2020–2021: *qap10510_qap10510*; 2022: *qUniekePatientenWmgHulpVerleend*; 2023: *qUniekePatientenWmgIngeschreven* | Annual number of patients treated per hospital | The source field changed due to revisions in the CIBG reporting structure. Year-specific patient-count fields were mapped to the same conceptual construct: annual hospital-level patients treated. |
| Patient days | 2017–2021: *qad10130_qad10130*, *qav11200_qav11200* | *patientdays = NumberCareDays + NumberNursingDays* | Available for 2017–2021. In 2022–2023, patient-day variables were no longer available in the CIBG reporting, therefore, LOS based on patient days could not be calculated for those years. |
| Length of stay | 2017–2021: *qad10130_qad10130, qav11200_qav11200, qap10510_qap10510* | LOS = patientdays / NumberPatientsTreated | Derived only where both patient days and patient-count data were available. Not calculated for 2022–2023 because patient-day fields were unavailable in the dataset. |
| Absenteeism | 2017–2021: *qPersTotLoonVerzuim_percVerzuim*; 2022 - 2023: *qConcPersWmgPersZorgVerzuim*; | Reported annual sickness absence percentage | Source fields changed over time. |
| Hospital size | 2017–2021: *qab09200_qab09200*; 2018–2019 also available as *qab09200Micro_qab09200*; 2022–2023: *qab17080_qab17080* | Number of hospital beds | Year-specific bed-capacity fields were harmonized into a single hospital-size variable. Missing bed values were inspected using descriptives and histograms. |
| Staff-to-patient-ratio | 2017: *qPersMsiLoonTot_AantalFte, qPersMsiZelfTot_AantalFte, qPersMsiHuurTot_AantalFte, qap10510_qap10510;* 2020–2021: *qPersTotTot_AantalPers*, *qap10510_qap10510*; 2022–2023: *qConcPersWmgPersTot*, | *Staff-to-patient ratio = total hospital staff / number of patients treated* | Staff variables were harmonized across years according to the available CIBG staff measure. Manual corrections were applied where source values were missing or inconsistent. Derived only when both staff and patient-count variables were available. |
| Teaching Status | *entMain_Typering_ent_Typering_0* or *UMC* | 1 = academic/UMC hospital; 0 = non-academic hospital |  |
| Digital maturity | HIMSS EMRAM validation information / public announcements; not derived from the CIBG cleaning syntax | 1 = HIMSS EMRAM Stage 6 or 7; 0 = Stage 0–5 or no public Stage 6/7 evidence | Assigned at the institutional-year level. Linkage to the CIBG hospital-year dataset was performed using hospital identity, name, and organizational identifier. |
| Year | Calendar year of the annual-report dataset | Continuous or categorical year variable, 2017–2023 | Included from the base longitudinal model onward to account for secular time trends. |
| Covid-Year | Calendar year | 1 = 2020–2021; 0 = otherwise | Sensitivity analysis can alternatively code 2020–2022 as COVID-affected years. |

1.	Tasi MC, Keswani A, Bozic KJ. Does physician leadership affect hospital quality, operational efficiency, and financial performance? Health Care Manage Rev. 2019 Jul/Sep;44(3):256-62. PMID: 28700509. doi: 10.1097/hmr.0000000000000173.

2.	Thiel A, Winter V, Büchner VA. Board characteristics, governance objectives, and hospital performance: An empirical analysis of German hospitals. Health Care Manage Rev. 2018 Oct/Dec;43(4):282-92. PMID: 28157831. doi: 10.1097/hmr.0000000000000153.

3.	Rosko M, Al-Amin M, Tavakoli M. Efficiency and profitability in US not-for-profit hospitals. Int J Health Econ Manag. 2020 Dec;20(4):359-79. PMID: 32816192. doi: 10.1007/s10754-020-09284-0.

4.	CIBG. Jaarverantwoording zorg. 2023 [cited 2025 07. July]; Available from: <https://digimv8.desan.nl/archive/search/results?year=2022&town=&organization>=.

5.	Collum TH, Menachemi N, Sen B. Does electronic health record use improve hospital financial performance? Evidence from panel data. Health Care Manage Rev. 2016 Jul-Sep;41(3):267-74. PMID: 26052785. doi: 10.1097/hmr.0000000000000068.

6.	Lalani K, Helton J, Vega FR, Cardenas-Turanzas M, Champagne-Langabeer T, Langabeer JR. The Impact of COVID-19 on the Financial Performance of Largest Teaching Hospitals. Healthcare (Basel). 2023 Jul 11;11(14). PMID: 37510437. doi: 10.3390/healthcare11141996.

7.	Dong GN. Earnings Management in U.S. Hospitals. Journal of Health and Human Services Administration. 2016;39(1):41-70. doi: 10.1177/107937391603900104.

8.	Dong GN. Performing well in financial management and quality of care: evidence from hospital process measures for treatment of cardiovascular disease. BMC Health Services Research. 2015 2015/02/01;15(1):45. doi: 10.1186/s12913-015-0690-x.

9.	van Poelgeest R, van Groningen JT, Daniels JH, Roes KC, Wiggers T, Wouters MW, et al. Level of Digitization in Dutch Hospitals and the Lengths of Stay of Patients with Colorectal Cancer. J Med Syst. 2017 May;41(5):84. PMID: 28391455. doi: 10.1007/s10916-017-0734-3.

10.	Hadji B, Meyer R, Melikeche S, Escalon S, Degoulet P. Assessing the relationships between hospital resources and activities: a systematic review. J Med Syst. 2014 Oct;38(10):127. PMID: 25171921. doi: 10.1007/s10916-014-0127-9.

11.	Gao H, Chen H, Feng J, Qin X, Wang X, Liang S, et al. Balanced scorecard-based performance evaluation of Chinese county hospitals in underdeveloped areas. Journal of International Medical Research. 2018;46(5):1947-62. PMID: 29562812. doi: 10.1177/0300060518757606.

12.	Flatow VH, Ibragimova N, Divino CM, Eshak DS, Twohig BC, Bassily-Marcus AM, et al. Quality Outcomes in the Surgical Intensive Care Unit after Electronic Health Record Implementation. Appl Clin Inform. 2015;6(4):611-8. PMID: 26767058. doi: 10.4338/aci-2015-04-ra-0044.

13.	Stone K, Zwiggelaar R, Jones P, Mac Parthaláin N. A systematic review of the prediction of hospital length of stay: Towards a unified framework. PLOS Digit Health. 2022 Apr;1(4):e0000017. PMID: 36812502. doi: 10.1371/journal.pdig.0000017.

14.	Maltezou HC, Ledda C, Sipsas NV. Absenteeism of Healthcare Personnel in the COVID-19 Era: A Systematic Review of the Literature and Implications for the Post-Pandemic Seasons. Healthcare (Basel). 2023 Nov 12;11(22). PMID: 37998442. doi: 10.3390/healthcare11222950.

15.	Millot C, Pereira B, Miallaret S, Clinchamps M, Vialatte L, Guillin A, et al. Statistical Analysis of Absenteeism in a University Hospital Center between 2007 and 2019. Int J Environ Res Public Health. 2022 Oct 10;19(19). PMID: 36232261. doi: 10.3390/ijerph191912966.

16.	HIMSS. Electronic Medical Record Adoption Model (EMRAM). 2024 [cited 2025 24.05]; Available from: <https://www.himss.org/maturity-models/emram/>.

17.	Snowdon A, Hussein A, Danforth M, Wright A, Oakes R. Digital Maturity as a Predictor of Quality and Safety Outcomes in US Hospitals: Cross-Sectional Observational Study. J Med Internet Res. 2024 Aug 6;26:e56316. PMID: 39106100. doi: 10.2196/56316.

18.	Giancotti M, Guglielmo A, Mauro M. Efficiency and optimal size of hospitals: Results of a systematic search. PLoS One. 2017;12(3):e0174533. PMID: 28355255. doi: 10.1371/journal.pone.0174533.

19.	McFarland DC, Ornstein KA, Holcombe RF. Demographic factors and hospital size predict patient satisfaction variance--implications for hospital value-based purchasing. J Hosp Med. 2015 Aug;10(8):503-9. PMID: 25940305. doi: 10.1002/jhm.2371.

20.	Butler M, Schultz TJ, Halligan P, Sheridan A, Kinsman L, Rotter T, et al. Hospital nurse-staffing models and patient- and staff-related outcomes. Cochrane Database Syst Rev. 2019 Apr 23;4(4):Cd007019. PMID: 31012954. doi: 10.1002/14651858.CD007019.pub3.

21.	McHugh MD, Berez J, Small DS. Hospitals with higher nurse staffing had lower odds of readmissions penalties than hospitals with lower staffing. Health Aff (Millwood). 2013 Oct;32(10):1740-7. PMID: 24101063. doi: 10.1377/hlthaff.2013.0613.

22.	Veiligheid Ov. Aanpak coronacrisis. Deel 3: januari 2020 – september 2022. Den Haag: Onderzoeksraad voor Veiligheid; 2023.
