# Supplementary material for: Impact of Digitalization on Performance of Dutch Hospitals: Longitudinal Quantitative Study
Source: J Med Internet Res. 2026 Sep 29;28:e94896. doi: 10.2196/94896 (PMC13662437; doi:10.2196/94896)
Supplement: Supplementary file 4 [file jmir_v28i1e94896_app4.docx]

| **Table 1 – Model descriptions** | |
| --- | --- |
| **Model** | **Description** |
| **Null Model** | Random intercept-only model (no predictors), used to estimate baseline ICC. |
| **Model 1** | Includes Year and Digitalization dummy |
| **Model 2** | Adds control variables: Teaching Status and Number of Beds to Model 1. |
| **Model 2.1** | Adds the Staff-to-Patient Ratio as a control variable to Model 2. |
| **Model 3** | Adds COVID-19 dummy to Model 2 (to control for pandemic effects). |
| **Model 3.1*** | Adds the Staff-to-Patient Ratio as a control variable to Model 3. |
| **Model 4** | Same as Model 2 but includes Year as a random effect. |
| **Model 4.1** | Adds Staff-to-Patient Ratio as a control variable to Model 4. |
| **Model 5** | Adds COVID-19 dummy to Model 4 (to control for pandemic effects). |
| **Model 5.1** | Adds Staff-to-Patient Ratio as a control variable to Model 4. |
| Note: * In manuscript reported Model | |

| **Table 2: Profit Margin – HIMSS EMRAM Mixed-effect Model development** | | | | | |
| --- | --- | --- | --- | --- | --- |
| **Parameter** | **Null Model** | **Model 1** | **Model 2** | **Model 2.1** | **Model 3** |
| **Intercept** | 1.979 (1.184- 2.775)*** | 1,683 (-3.175- 6.540) | -87.435 (-402.069- 227.200) | -71.956 (-395.970- 252.059) | -119.657 (-434.013- 194.698) |
| ***Control Variables*** | | | | | |
| **Academic hospital** |  |  | -0.142 (-1.517- 1.234) | -0.140 (-1.533- 1.253) | -0.114 (-1.481- 1.253) |
| **Size (Number of Beds)** |  |  | -0.001 (-0.005- 0.002) | 0.001 (-0.002- 0.003) | 0.000 (-0.002- 0.003) |
| **Staff-to-Patient Ratio** |  |  |  | 2.024 (-3.673- 7.722) |  |
| ***Independent Variables*** | | | | | |
| **Year** |  | 0.035 (-0.119- 0.189) | 0.044 (-0.112- 0.200) | 0.036 (-0.124- 0.197) | 0.060 (-0.095- 0.216) |
| **COVID Year** |  |  |  |  | -0.458 (-0.818- -0.098)* |
| **Dummy high degree of digitalization** |  | -0.251 (-1-917- 1.416) | -0.311 (-1.989- 1.366) | 0.033 (-1.733- 1.799) | -0.220 (-1.893- 1.453) |
| **ICC** | 0.43 | 0.42 | 0.40 | 0.39 | 0.40 |
| **AIC** | 2176.386 | 2180.113 | 2171.447 | 2113.7491 | 2167.217 |
| **BIC** | 2193.172 | 2205.29 | 2204.970 | 2151.123 | 2204.930 |
| *Note:* Coefficients are reported with 95% confidence intervals in parentheses. * *p* < 0.05, ** p < 0.01, *** p < 0.001.  *Note:* Variance-Inflation Factors (VIF) are reported in Appendix F. All VIF values were below the threshold of 5 (mean = 1.8), indicating no relevant multicollinearity. | | | | |  |

| **Table 2: Profit Margin – HIMSS EMRAM Mixed-effect Model development** | | | | | |
| --- | --- | --- | --- | --- | --- |
| **Parameter** | **Model 3.1** | **Model 4** | **Model 4.1** | **Model 5** | **Model 5.1** |
| **Intercept** | -104.924 (-429.051- 219.203) | -86.922 (-400.518- 226.674) | -71.352 (-394.258- 251.553) | -119.204 (-432.515- 194.108) | -104.369 (-427.370- 218.631) |
| ***Control Variables*** | | | | | |
| **Academic hospital** | -0.109 (-1.494- 1.275) | -0.143 (-1.517- 1.232) | -0.141 (-1.533- 1.252) | -0.115 (-1.482- 1.252) | -0.110 (-1.494- 1.274) |
| **Size (Number of Beds)** | 0.000 (-0.002- 0.003) | 0.001 (-0.002- 0.003) | 0.001 (-0.002- 0.003) | 0.000 (-0.002- 0.003) | 0.000 (-0.002- 0.003) |
| **Staff-to-Patient Ratio** | 2.073 (-3.582-7.728) |  | 2.026 (-3.671- 7.723) |  | 2.075 (-3.580- 7.730) |
| ***Independent Variables*** | | | | | |
| **Year** | 0.053 (-0.108- 0.213) | 0.044 (-0.111- 0.199) | 0.036 (-0.124- 0.196) | 0.060 (-0.095- 0.215) | 0.053 (-0.107- 0.212) |
| **COVID Year** | -0.463 (-0.835- -0.092)* |  |  | -0.458 (-0.818- -0.098)* | -0.463 (-0.835- -0.092)* |
| **Dummy high degree of digitalization** | 0.098 (-1.662- 1.859) | -0.310 (-1.989- 1.368) | -0.025 (-0.428- 0.379) | -0.219 (-1.893- 1.454) | 0.100 (-1.661- 1.861) |
| **ICC** | 0.38 | 0.00 | 0.00 | 0.00 | 0.00 |
| **AIC** | 2109.774 | 2173.327 | 2115.635 | 2169.098 | 2111.663 |
| **BIC** | 2151.302 | 2211.040 | 2157.163 | 2211.001 | 2157.343 |
| *Note:* Coefficients are reported with 95% confidence intervals in parentheses. * *p* < 0.05, ** p < 0.01, *** p < 0.001.  *Note:* Variance-Inflation Factors (VIF) are reported in Appendix F. All VIF values were below the threshold of 5 (mean = 1.8), indicating no relevant multicollinearity. | | | | |  |

| **Table 3: Return on Assets (ROA) – HIMSS EMRAM Mixed-effect Model development** | | | | | |
| --- | --- | --- | --- | --- | --- |
| **Parameter** | **Null Model** | **Model 1** | **Model 2** | **Model 2.1** | **Model 3** |
| **Intercept** | 0.159 (-0.113- 0.430) | -143.024 (-416.323- 130.276) | -154.655 (-440.331- 131.021) | -173.490 (-470.721- 123.741) | -178.642 (-470.734- 113.449) |
| ***Control Variables*** | | | | | |
| **Academic hospital** |  |  | 1.531 (0.502- 2.559)** | 1.785 (0.689- 2.881)** | 1.589 (0.546- 2.633)** |
| **Size (Number of Beds)** |  |  | -0.000 (-0.001- 0.001) | 0.000 (-0.001- 0.001) | 0.000 (-0.001- 0.001) |
| **Staff-to-Patient Ratio** |  |  |  | -5.707 (-14.172- 2.759) |  |
| ***Independent Variables*** | | | | | |
| **Year** |  | 0.071 (-0.061- 0.206) | 0.077 (-0.065- 0.218) | 0.086 (-0.061- 0.233) | 0.089 (-0.056- 0.233) |
| **COVID Year** |  |  |  |  | -0.291 (-0.898- 0.315) |
| **Dummy high degree of digitalization** |  | -0.187 (-0.904- 0.531) | -0.355 (-1.173- 0.464) | -0.384 (-1.231- 0.462) | -0.335 (-1.189- 0.478) |
| **ICC** | 0.00 | 0.00 | 0.01 | 0.01 | 0.01 |
| **AIC** | 2490.092 | 2492.785 | 2475.391 | 2402.275 | 2476.524 |
| **BIC** | 2506.886 | 2517.976 | 2508.929 | 2439.668 | 2514.255 |
| *Note:* Coefficients are reported with 95% confidence intervals in parentheses. * *p* < 0.05, ** p < 0.01, *** p < 0.001.  *Note:* Variance-Inflation Factors (VIF) are reported in Appendix F. All VIF values were below the threshold of 5 (mean = 1.8), indicating no relevant multicollinearity. | | | | |  |

| **Table 3: Return on Assets (ROA) – HIMSS EMRAM Mixed-effect Model development** | | | | | |
| --- | --- | --- | --- | --- | --- |
| **Parameter** | **Model 3.1** | **Model 4** | **Model 4.1** | **Model 5** | **Model 5.1** |
| **Intercept** | -198.530 (-501.842- 104.782) | -154.721 (-440.288- 130.288) | -173.583 (-470.741- 123.576) | -178.702 (-470.642- 113.239) | -198.561 (-501.891- 104.590) |
| ***Control Variables*** | | | | | |
| **Academic hospital** | 1.840 (0.731- 2.949) | 1.534 (0.505- 2.563)** | 1.789 (0.691- 2.886)** | 1.591 (0.548- 2.635)** | 1.844 (0.734- 2.953) |
| **Size (Number of Beds)** | -0.000 (-0.001- 0.001) | -0.000 (-0.001- 0.001) | -0.000 (-0.001- 0.001) | -0.000 (-0.001- 0.001) | -0.000 (-0.001- 0.001) |
| **Staff-to-Patient Ratio** | -5.721 (-14.198- 2.755) |  | -5.712 (-14.178- 2.755) |  | -5.726 (-14.204- 2.751) |
| ***Independent Variables*** | | | | | |
| **Year** | 0.098 (-0.052- 0.249) | 0.077 (-0.065- 0.218) | 0.086 (-0.061- 0.233) | 0.089 (-0.056- 0.233) | 0.099 (-0.052- 0.249) |
| **COVID Year** | -0.307 (-0.935- 0.320) |  |  | -0.291 (-0.898- 0.315) | -0.307 (-0.935- 0.320) |
| **Dummy high degree of digitalization** | -0.381 (-1.240- 0.478) | -0.355 (-1.174- 0.465) | -0.384 (-1.232- 0.463) | -0.355 (-1.189- 0.478) | -0.381 (-1.241- 0.479) |
| **ICC** | 0.02 | 0.00 | 0.00 | 0.00 | 0.00 |
| **AIC** | 2403.371 | 2477.375 | 2404.260 | 2478.510 | 2405.356 |
| **BIC** | 2444.920 | 2515.108 | 2445.809 | 2520.433 | 2415.060 |
| *Note:* Coefficients are reported with 95% confidence intervals in parentheses. * *p* < 0.05, ** p < 0.01, *** p < 0.001.  *Note:* Variance-Inflation Factors (VIF) are reported in Appendix F. All VIF values were below the threshold of 5 (mean = 1.8), indicating no relevant multicollinearity. | | | | |  |

| **Table 4: Personnel-Expense-Ratio (PER) – HIMSS EMRAM Mixed-effect Model development** | | | | | |
| --- | --- | --- | --- | --- | --- |
| **Parameter** | **Null Model** | **Model 1** | **Model 2** | **Model 2.1** | **Model 3** |
| **Intercept** | 48.909 (47.634- 50.185)*** | 1480.108 (785.485- 2174- 2174.731)*** | 1441.654 (757.980- 2125.327)*** | 1389.888 (695.147- 2084.629)*** | 1626.806 (969.405- 2284.207)*** |
| ***Control Variables*** | | | | | |
| **Academic hospital** |  |  | 4.229 (1.432- 7.026)** | 4.115 (1.298- 6.933)** | 3.784 (1.094- 6.474)** |
| **Size (Number of Beds)** |  |  | -0.001 (-0.005- 0.003) | 0.000 (-0.004- 0.003) | 0.001 (-0.003- 0.004) |
| **Staff-to-Patient Ratio** |  |  |  | -0.182 (-12.954- 12.590) |  |
| ***Independent Variables*** | | | | | |
| **Year** |  | -0.709 (-1.052- -0.365)*** | -0.690 (-1.028- -0.351)*** | -0.664 (-1.008- -0.320)*** | -0.782 (-1.107- -0.456)*** |
| **COVID Year** |  |  |  |  | -0.291 (-0.898- 0.315) |
| **Dummy high degree of digitalization** |  | 1.687 (-1.249- 4.624) | 1.375 (-1.448- 4.197) | 1.526 (-1.378- 4.429) | 3.026 (2.232- 3.820)*** |
| **ICC** | 0.00 | 0.00 | 0.00 | 0.00 | 0.00 |
| **AIC** | 2926.169 | 2913.262 | 2892.081 | 2798.615 | 2841.676 |
| **BIC** | 2942.914 | 2938.379 | 2925.521 | 2835.913 | 2879.296 |
| *Note:* Coefficients are reported with 95% confidence intervals in parentheses. * *p* < 0.05, ** p < 0.01, *** p < 0.001.  *Note:* Variance-Inflation Factors (VIF) are reported in Appendix F. All VIF values were below the threshold of 5 (mean = 1.8), indicating no relevant multicollinearity. | | | | |  |

| **Table 4: Personnel-Expense-Ratio (PER) – HIMSS EMRAM Mixed-effect Model development** | | | | | |
| --- | --- | --- | --- | --- | --- |
| **Parameter** | **Model 3.1** | **Model 4** | **Model 4.1** | **Model 5** | **Model 5.1** |
| **Intercept** | 1580.374 (914.730- 2246.017)*** | 1441.654 (757.980- 2125.327)*** | 1389.888 (695.147- 2084.629)*** | 1626.806 (969.405- 2284.207)*** | 1580.374 (914.730- 2246.017)*** |
| ***Control Variables*** | | | | | |
| **Academic hospital** | 3.629 (0.930- 6.327)** | 4.229 (1.432- 7.026)** | 4.115 (1.298- 6.933)** | 3.784 (1.094- 3.820)** | 3.629 (0.930- 6.327)** |
| **Size (Number of Beds)** | 0.001 (-0.003- 0.005) | -0.001 (-0.005- 0.003) | 0.000 (-0.004- 0.003) | 0.000 (-0.003- 0.004) | 0.000 (-0.003- 0.005) |
| **Staff-to-Patient Ratio** | -1.068 (-12.987- 10.851) |  | -0.182 (-12.954- 12.590) |  | -1.068 (-12.987- 10.851) |
| ***Independent Variables*** | | | | | |
| **Year** | -0.759 (-1.088- -0.429)*** | -0.690 (-1.028- -0.351)*** | -0.664 (-1.008- -0.320)*** | -0.782 (-1.107- -0.456)*** | -0.759 (-1.088- -0.429)*** |
| **COVID Year** | 3.135 (2.333- 3.937)*** |  |  | -0.291 (-0.898- 0.315) | 3.135 (2.333- 3.937)*** |
| **Dummy high degree of digitalization** | 1.340 (-1.544- 4.225) | 1.375 (-1.448- 4.197) | 1.526 (-1.378- 4.429) | 3.026 (2.232- 3.820)*** | 1.340 (-1.544- 4.225) |
| **ICC** | 0.00 | 0.00 | 0.00 | 0.00 | 0.00 |
| **AIC** | 2745.881 | 2894.081 | 2800.615 | 2843.676 | 2747.881 |
| **BIC** | 2787.323 | 2931.701 | 2842.057 | 2885.476 | 2793.467 |
| *Note:* Coefficients are reported with 95% confidence intervals in parentheses. * *p* < 0.05, ** p < 0.01, *** p < 0.001.  *Note:* Variance-Inflation Factors (VIF) are reported in Appendix F. All VIF values were below the threshold of 5 (mean = 1.8), indicating no relevant multicollinearity. | | | | |  |

| **Table 5: Asset-Turnover-Ratio (ATR) – HIMSS EMRAM Mixed-effect Model development** | | | | | |
| --- | --- | --- | --- | --- | --- |
| **Parameter** | **Null Model** | **Model 1** | **Model 2** | **Model 2.1** | **Model 3** |
| **Intercept** | 0.053 (0.027- 0.079)*** | 14.926 (-11.530- 41.381) | 15.180 (-11.441- 41.802) | 14.782 (11.256- 18.308)*** | 18.399 (-8.580- 45.378) |
| ***Control Variables*** | | | | | |
| **Academic hospital** |  |  | 0.028 (-0.063- 0.118) | 0.017 (0.004- 0.030)* | 0.027 (-0.063- 0.118) |
| **Size (Number of Beds)** |  |  | 0.000 (0.000- 0.000) | 0.000 (0.000- 0.000) | 0.000 (0.000- 0.000) |
| **Staff-to-Patient Ratio** |  |  |  | 0.008 (-0.076- 0.092) |  |
| ***Independent Variables*** | | | | | |
| **Year** | v | -0.709 (-1.052- -0.365)*** | -0.690 (-1.028- -0.351)*** | -0.664 (-1.008- -0.320)*** | -0.782 (-1.107- -0.456)*** |
| **COVID Year** |  |  |  |  | -0.291 (-0.898- 0.315) |
| **Dummy high degree of digitalization** |  | 1.687 (-1.249- 4.624) | 1.375 (-1.448- 4.197) | 1.526 (-1.378- 4.429) | 3.026 (2.232- 3.820)*** |
| **ICC** | 0.00 | 0.00 | 0.00 | 0.00 | 0.00 |
| **AIC** | 2926.169 | 2913.262 | 2892.081 | 2798.615 | 2841.676 |
| **BIC** | 2942.914 | 2938.379 | 2925.521 | 2835.913 | 2879.296 |
| *Note:* Coefficients are reported with 95% confidence intervals in parentheses. * *p* < 0.05, ** p < 0.01, *** p < 0.001.  *Note:* Variance-Inflation Factors (VIF) are reported in Appendix F. All VIF values were below the threshold of 5 (mean = 1.8), indicating no relevant multicollinearity. | | | | |  |

| **Table 5: Asset-Turnover-Ratio (ATR) – HIMSS EMRAM Mixed-effect Model development** | | | | | |
| --- | --- | --- | --- | --- | --- |
| **Parameter** | **Model 3.1** | **Model 4** | **Model 4.1** | **Model 5** | **Model 5.1** |
| **Intercept** | 14.711 (11.171- 18.250)*** | 15.180 (-11.441- 41.802) | 14.782 (11.256- 18.308)*** | 18.399 (-8.580- 45.378) | 14.711 (11.171- 18.250)*** |
| ***Control Variables*** | | | | | |
| **Academic hospital** | 0.017 (0.004- 0.030)* | 0.028 (-0.063- 0.118) | 0.017 (0.004- 0.030) | 0.027 (-0.063- 0.118) | 0.017 (0.004- 0.030)* |
| **Size (Number of Beds)** | 0.000 (0.000- 0.000) | 0.000 (0.000- 0.000) | 0.000 (0.000- 0.000) | 0.000 (0.000- 0.000) | 0.000 (0.000- 0.000) |
| **Staff-to-Patient Ratio** | 0.008 (-0.076- 0.092) |  | 0.008 (-0.076- 0.092) |  | 0.008 (-0.076- 0.092) |
| ***Independent Variables*** | | | | | |
| **Year** | -0.007 (-0.009- -0.006)*** | -0.007 (-0.021- 0.006) | -0.007 (-0.009- -0.006) | -0.009 (-0.022- 0.004) | -0.007 (-0.003- -0.006) |
| **COVID Year** | -0.001 (-0.007- 0.005) |  |  | 0.040 (-0.018- 0.099) | -0.001 (-0.007- 0.005) |
| **Dummy high degree of digitalization** | 0.008 (-0.003- 0.019) | -0.002 (-0.073- 0.069) | 0.008 (-0.003- 0.019) | -0.002 (-0.073- 0.068) | 0.008 (-0.003- 0.019) |
| **ICC** | 0.00 | 0.00 | 0.00 | 0.00 | 0.00 |
| **AIC** | -1973.938 | 207.461 | -1953.784 | 207.637 | -1951.363 |
| **BIC** | -1912.389 | 245.174 | -1912.236 | 249.540 | -1906.234 |
| *Note:* Coefficients are reported with 95% confidence intervals in parentheses. * *p* < 0.05, ** p < 0.01, *** p < 0.001.  *Note:* Variance-Inflation Factors (VIF) are reported in Appendix F. All VIF values were below the threshold of 5 (mean = 1.8), indicating no relevant multicollinearity. | | | | |  |

| **Table 6: Number of Patients Treated – HIMSS EMRAM Mixed-effect Model development** | | | | | |
| --- | --- | --- | --- | --- | --- |
| **Parameter** | **Null Model** | **Model 1** | **Model 2** | **Model 2.1** | **Model 3** |
| **Intercept** | 137317.969 (120441.864- 154194.074)*** | 126808.817 (109480.081- 144137.554)*** | 27171.176 (7614.327- 46728.030)** | 50981.969 (30833.951- 71129.986)*** | 32144.408 (12261.289- 52027.527)** |
| ***Control Variables*** | | | | | |
| **Academic hospital** |  |  | -46776.972 (-77340.813- -16213.131)** | -27780.010 (-57951.472- 2391.451) | -46932.168 (-77229.908- -16634.428)** |
| **Size (Number of Beds)** |  |  | 206.563 (170.497- 242.630)*** | 202.932 (168.214- 237.650)*** | 205.110 (169.360- 240.861)*** |
| **Staff-to-Patient Ratio** |  |  |  | -661152.348 (-863846.240- -458458.457)*** |  |
| ***Independent Variables*** | | | | | |
| **Year** |  | 0.001 (.) | 0.002 (.) | 0.002 (.) | 0.002 (.) |
| **COVID Year** |  |  |  |  | 0.040 (-0.018- 0.099) |
| **Dummy high degree of digitalization** |  | 59885.465 (20040.956- 99729.975)** | 38748.225 (13612.725- 63883.726)** | 37380.559 (13140.125- 61620.993)** | -16920.640 (-32258.644—1582.636)* |
| **ICC** | 0.27 | 0.20 | 0.00 | 0.00 | 0.00 |
| **AIC** | 12241.054 | 12244.789 | 12103.145 | 11943.919 | 12100.166 |
| **BIC** | 12265.758 | 12269.844 | 12136.195 | 11981.351 | 12137.693 |
| *Note:* Coefficients are reported with 95% confidence intervals in parentheses. * *p* < 0.05, ** p < 0.01, *** p < 0.001.  *Note:* Variance-Inflation Factors (VIF) are reported in Appendix F. All VIF values were below the threshold of 5 (mean = 1.8), indicating no relevant multicollinearity. | | | | |  |

| **Table 6: Number of Patients Treated – HIMSS EMRAM Mixed-effect Model development** | | | | | |
| --- | --- | --- | --- | --- | --- |
| **Parameter** | **Model 3.1** | **Model 4** | **Model 4.1** | **Model 5** | **Model 5.1** |
| **Intercept** | 55444.524 (35014.686- 75874.361)*** | 26606.329 (8567.188- 44645.471)** | 50981.969 (30833.950- 71129.989)*** | 32144.408 (12261.289- 52027.527)** | 55444.524 (35014.688- 75874.360)*** |
| ***Control Variables*** | | | | | |
| **Academic hospital** | -27979.696 (-57925.124- 1965.733) | -48038.612 (-76483.805- -19593.420)** | -27780.010 (-57951.474- 2391.454) | -46932.168 (-77229.908- -16634.428)** | -27979.696 (-57925.122- 1965.730) |
| **Size (Number of Beds)** | 201.570 (167.116- 236.024)*** | 207.519 (174.153- 240.886)*** | 202.932 (168.214- 237.650)*** | 205.110 (169.360- 240.861)*** | 201.570 (167.116- 236.024)*** |
| **Staff-to-Patient Ratio** | -654830.627 (-856678.245- -452983.009)*** |  | -661152.349 (-863846.243- -458458.454)*** |  | -654830.627 (-856678.24- -452983.012)*** |
| ***Independent Variables*** | | | | | |
| **Year** | 0.002 (.) | 0.002 (.) | 0.002 (.) | 0.002 (.) | 0.002 (.) |
| **COVID Year** | -15662.722 (-30501.711- -823.733)* |  |  | -16920.640 (-32258.644- -1582.636)* | -15662.722 (-30501.711- -823.733)* |
| **Dummy high degree of digitalization** | 37842.441 (13808.284- 61876.598)** | 38310.022 (15092.139- 61527.905)** | 37380.559 (13140.122- 61620.996)** | 39359.201 (14463.345- 64255.057)** | 37842.441 (13808.286- 61876.596)** |
| **ICC** | 0.00 | 0.00 | 0.00 | 0.00 | 0.00 |
| **AIC** | 11941.640 | 12107.207 | 11945.919 | 12102.166 | 11943.640 |
| **BIC** | 11983.231 | 12144.733 | 11987.510 | 12143.863 | 11989.390 |
| *Note:* Coefficients are reported with 95% confidence intervals in parentheses. * *p* < 0.05, ** p < 0.01, *** p < 0.001.  *Note:* Variance-Inflation Factors (VIF) are reported in Appendix F. All VIF values were below the threshold of 5 (mean = 1.8), indicating no relevant multicollinearity. | | | | |  |

| **Table 6: Number of Patients Treated – HIMSS EMRAM Mixed-effect Model development** | | | | | |
| --- | --- | --- | --- | --- | --- |
| **Parameter** | **Model 3.1** | **Model 4** | **Model 4.1** | **Model 5** | **Model 5.1** |
| **Intercept** | 55444.524 (35014.686- 75874.361)*** | 26606.329 (8567.188- 44645.471)** | 50981.969 (30833.950- 71129.989)*** | 32144.408 (12261.289- 52027.527)** | 55444.524 (35014.688- 75874.360)*** |
| ***Control Variables*** | | | | | |
| **Academic hospital** | -27979.696 (-57925.124- 1965.733) | -48038.612 (-76483.805- -19593.420)** | -27780.010 (-57951.474- 2391.454) | -46932.168 (-77229.908- -16634.428)** | -27979.696 (-57925.122- 1965.730) |
| **Size (Number of Beds)** | 201.570 (167.116- 236.024)*** | 207.519 (174.153- 240.886)*** | 202.932 (168.214- 237.650)*** | 205.110 (169.360- 240.861)*** | 201.570 (167.116- 236.024)*** |
| **Staff-to-Patient Ratio** | -654830.627 (-856678.245- -452983.009)*** |  | -661152.349 (-863846.243- -458458.454)*** |  | -654830.627 (-856678.24- -452983.012)*** |
| ***Independent Variables*** | | | | | |
| **Year** | 0.002 (.) | 0.002 (.) | 0.002 (.) | 0.002 (.) | 0.002 (.) |
| **COVID Year** | -15662.722 (-30501.711- -823.733)* |  |  | -16920.640 (-32258.644- -1582.636)* | -15662.722 (-30501.711- -823.733)* |
| **Dummy high degree of digitalization** | 37842.441 (13808.284- 61876.598)** | 38310.022 (15092.139- 61527.905)** | 37380.559 (13140.122- 61620.996)** | 39359.201 (14463.345- 64255.057)** | 37842.441 (13808.286- 61876.596)** |
| **ICC** | 0.00 | 0.00 | 0.00 | 0.00 | 0.00 |
| **AIC** | 11941.640 | 12107.207 | 11945.919 | 12102.166 | 11943.640 |
| **BIC** | 11983.231 | 12144.733 | 11987.510 | 12143.863 | 11989.390 |
| *Note:* Coefficients are reported with 95% confidence intervals in parentheses. * *p* < 0.05, ** p < 0.01, *** p < 0.001.  *Note:* Variance-Inflation Factors (VIF) are reported in Appendix F. All VIF values were below the threshold of 5 (mean = 1.8), indicating no relevant multicollinearity. | | | | |  |

| **Table 7: Length of Stay (LOS) – HIMSS EMRAM Mixed-effect Model development** | | | | | |
| --- | --- | --- | --- | --- | --- |
|  | | | | | |
| **Parameter** | **Null Model** | **Model 1** | **Model 2** | **Model 2.1** | **Model 3** |
| **Intercept** | 1.159 (0.915- 1.404)*** | 71.791 45.321- 98.261)*** | 72.594 (44.539- 100.648)*** | 158.148 (122.803- 193.494)*** | 36.827 (-34.796- 108.450) |
| ***Control Variables*** | | | | | |
| **Academic hospital** |  |  | -0.032 (-0.443- 0.379) | -0.156 (-0.549- 0.237) | -0.040 (-0.450- 0.371) |
| **Size (Number of Beds)** |  |  | 0.000 (0.000- 0.000) | 0.000 (0.000- 0.000) | 0.000 (0.000- 0.000) |
| **Staff-to-Patient Ratio** |  |  |  | 15.653 (13.783- 17.522)*** |  |
| ***Independent Variables*** | | | | | |
| **Year** |  | -0.035 (-0.048- -0.022)*** | -0.035 (-0.049- -0.022)*** | -0.078 (-0.095- -0.061)*** | -0.018 (-0.053- 0.018) |
| **COVID Year** |  |  |  |  | -0.053 (-0.152- 0.045) |
| **Dummy high degree of digitalization** |  | -0.037 (-0.268- 0.194) | -0.036 (-0.267- 0.196) | -0.126 (-0.424- 0.172) | -0.035 (-0.266- 0.196) |
| **ICC** | 0.92 | 0.93 | 0.93 | 0.96 | 0.93 |
| **AIC** | 392.906 | 370.505 | 374.207 | 245.631 | 375.066 |
| **BIC** | 408.221 | 393.227 | 404.839 | 279.985 | 409.527 |
| *Note:* Coefficients are reported with 95% confidence intervals in parentheses. * *p* < 0.05, ** p < 0.01, *** p < 0.001.  *Note:* Variance-Inflation Factors (VIF) are reported in Appendix F. All VIF values were below the threshold of 5 (mean = 1.8), indicating no relevant multicollinearity. | | | | |  |

| **Table 7: Length of Stay – HIMSS EMRAM Mixed-effect Model development** | | | | | |
| --- | --- | --- | --- | --- | --- |
| **Parameter** | **Model 3.1** | **Model 4** | **Model 4.1** | **Model 5** | **Model 5.1** |
| **Intercept** | 113.075 (48.779- 177.371)*** | 72.632 (44.555- 100.709)*** | 158.199 (122.832- 193.567)*** | 36.853 (-34.821- 108.527) | 113.049 (48.766- 177.331)*** |
| ***Control Variables*** | | | | | |
| **Academic hospital** | -0.154 (-0.548- 0.239) | 0.028 (-0.063- 0.118) | -0.156 (-0.549- 0.237) | -0.041 (-0.451- 0.369) | -0.154 (-0.547- 0.240) |
| **Size (Number of Beds)** | 0.000 (0.000- 0.000) | 0.000 (0.000- 0.000) | 0.000 (0.000- 0.000) | 0.000 (0.000- 0.000) | 0.000 (0.000- 0.000) |
| **Staff-to-Patient Ratio** | 15.791 (13.944- 17.639)*** |  | 15.663 (13.794- 17.532)*** |  | 15.801 (13.954-17.649)*** |
| ***Independent Variables*** | | | | | |
| **Year** | -0.056 (-0.088- -0.024)*** | -0.035 (-0.049- -0.022)*** | -0.078 (-0.096- -0.061)*** | -0.,018 (-0.053- 0.018) | -0.056 (-0.087- -0.024)*** |
| **COVID Year** | -0.077 (-0.167- 0.014) |  |  | -0.054 (-0.152- 0.045) | -0.077 (-0.167- 0.014) |
| **Dummy high degree of digitalization** | -0.126 (-0.425- 0.173) | -0.035 (-0.267- 0.196) | -0.126 (-0.424- 0.172) | -0.034 (-0.266-0.197) | -0.126 (-0.425- 0.174) |
| **ICC** | 0.96 | 0.00 | 0.00 | 0.00 | 0.00 |
| **AIC** | 245.028 | 376.447 | 247.603 | 377.307 | 246.987 |
| **BIC** | 283.199 | 410.907 | 285.774 | 415.596 | 288.975 |
| *Note:* Coefficients are reported with 95% confidence intervals in parentheses. * *p* < 0.05, ** p < 0.01, *** p < 0.001.  *Note:* Variance-Inflation Factors (VIF) are reported in Appendix F. All VIF values were below the threshold of 5 (mean = 1.8), indicating no relevant multicollinearity. | | | | |  |

| **Table 8: Absenteeism – HIMSS EMRAM Mixed-effect Model development** | | | | | |
| --- | --- | --- | --- | --- | --- |
|  | | | | | |
| **Parameter** | **Null Model** | **Model 1** | **Model 2** | **Model 2.1** | **Model 3** |
| **Intercept** | 5.785 (5.565- 6.004)*** | -697.374 (-772.738- -610.011)*** | -694.452 (-776.388- -612.516)*** | -683.224 (-772.532- -593.915)*** | -695.480 (-779.684- -611.277)*** |
| ***Control Variables*** | | | | | |
| **Academic hospital** |  |  | -0.151 (-0.607- 0.305) | -0.272 (-0.747- 0.202) | -0.151 (-0.806- 0.121) |
| **Size (Number of Beds)** |  |  | 0.000 (0.000- 0.000) | -0.000 (-0.00- 0.001) | -0.000 (-0.001- 0.001) |
| **Staff-to-Patient Ratio** |  |  |  | 0.363 (-2.417- 3.143) |  |
| ***Independent Variables*** | | | | | |
| **Year** |  | 0.345 (0.305- 0.385)*** | 0.347 (0.306- 0.387)*** | 0.341 (0.297- 0.385)*** | 0.347 (0.306- 0.389)*** |
| **COVID Year** |  |  |  |  | -0.011 (-0.185- 0.163) |
| **Dummy high degree of digitalization** |  | -0.360 (-0.825- 0.104) | -0.344 (-0.807- 0.120) | -0.381 (-0.849- 0.088) | -0.342 (-0.806- 0.121) |
| **ICC** | 0.12 | 0.49 | 0.48 | 0.44 | 0.48 |
| **AIC** | 1528.588 | 1402.505 | 1395.015 | 1351.554 | 1397.000 |
| **BIC** | 1545.390 | 1427.708 | 1425.570 | 1388.967 | 1434.750 |
| *Note:* Coefficients are reported with 95% confidence intervals in parentheses. * *p* < 0.05, ** p < 0.01, *** p < 0.001.  *Note:* Variance-Inflation Factors (VIF) are reported in Appendix F. All VIF values were below the threshold of 5 (mean = 1.8), indicating no relevant multicollinearity. | | | | |  |

| **Table 8: Absenteeism – HIMSS EMRAM Mixed-effect Model development** | | | | | |
| --- | --- | --- | --- | --- | --- |
| **Parameter** | **Model 3.1** | **Model 4** | **Model 4.1** | **Model 5** | **Model 5.1** |
| **Intercept** | -688.171 (-779.927- -596.415)*** | -694.541 (-776.413- -612.669)*** | -683.311 (-772.547- -594.074)*** | -695.554 (-779.695- -611.414)*** | -688.247 (-779.932- -596.563)*** |
| ***Control Variables*** | | | | | |
| **Academic hospital** | -0.277 (-0.752- 0,198) | -0.150 (-0.606- 0.306) | -0.272 (-0.746- 0.202) | -0.151 (-0.607- 0.305) | -0.276 (-0.751- 0.198) |
| **Size (Number of Beds)** | -0.000 (-0.001- 0.001) | -0.000 (-0.001- 0.001) | -0.000 (-0.001- 0.001) | -0.000 (-0.001- 0.001) | -0.000 (-0.001- 0.001) |
| **Staff-to-Patient Ratio** | 0.359 (-2.422-3.139) |  | 0.364 (-2.416- 3.143) |  | 0.359 (-2.421- 3.139) |
| ***Independent Variables*** | | | | | |
| **Year** | 0.344 (0.298- 0.389)*** | 0.347 (0.306- 0.387)*** | 0.341 (0.297- 0.385)*** | 0.347 (0.306- 0.389)*** | 0.344 (0.398- 0.389)*** |
| **COVID Year** | -0.056 (-0.243- 0.124) |  |  | -0.011 (-0.184- 0.163) | -0.059 (-0.242- 0.124) |
| **Dummy high degree of digitalization** | -0.375 (-0.844- 0.093) | -0.343 (-0.806- 0.121) | -0.380 (-0.849- 0.0888) | 0.341 (-0.805- 0.122) | -0.375 (-0.843- 0.093) |
| **ICC** | 0.44 | 0.00 | 0.00 | 0.00 | 0.00 |
| **AIC** | 1353.161 | 1396.832 | 1353.392 | 1398.817 | 1355.002 |
| **BIC** | 1394.731 | 1434.582 | 1394.962 | 1440.762 | 1400.729 |
| *Note:* Coefficients are reported with 95% confidence intervals in parentheses. * *p* < 0.05, ** p < 0.01, *** p < 0.001.  *Note:* Variance-Inflation Factors (VIF) are reported in Appendix F. All VIF values were below the threshold of 5 (mean = 1.8), indicating no relevant multicollinearity. | | | | |  |
