# Supplementary material for: Impact of Digitalization on Performance of Dutch Hospitals: Longitudinal Quantitative Study
Source: J Med Internet Res. 2026 Sep 29;28:e94896. doi: 10.2196/94896 (PMC13662437; doi:10.2196/94896)
Supplement: Supplementary file 5 [file jmir_v28i1e94896_app5.docx]

| **Comparison of Hospital Characteristics by HIMSS EMRAM Stage: Stages ≤5 vs Stages 6–7** | | | | |
| --- | --- | --- | --- | --- |
|  | **EMRAM Stages 5 - 0** | | ***EMRAM Stages 6 and 7*** | |
| **Variables** | **N** | ***Mean (SD)*** | ***N*** | ***Mean (SD)*** |
| Number of Beds | 406 | 483.84 (228.27) | 81 | 618.16 (202.28) |
| Teaching Status | 409 | 0.09 (0.29) | 81 | 0.20 (0.40) |
| Staff Employed | 404 | 3613.85 (2826.18) | 81 | 5537.99 (3171.96) |
| Staff-to-patient-ratio | 395 | 0.036 (0.037) | 78 | 0.036 (0.022) |
| Number of Patients Treated | 400 | 120417.37 (87131.07) | 78 | 179408.31 (124535.41) |
|  |  |  |  |  |
|  |  |  |  |  |
|  |  |  |  |  |
|  |  |  |  |  |
|  |  |  |  |  |
|  |  |  |  |  |
|  |  |  |  |  |
|  |  |  |  |  |
