# Supplementary material for: Impact of Digitalization on Performance of Dutch Hospitals: Longitudinal Quantitative Study
Source: J Med Internet Res. 2026 Sep 29;28:e94896. doi: 10.2196/94896 (PMC13662437; doi:10.2196/94896)
Supplement: Supplementary file 6 [file jmir_v28i1e94896_app6.docx]

**Variance-Inflation-Factor-Analysis**

| **Outcome Variable** | **Profit Margin** | | **Return on Assets** | | **Personnel Expense Rato** | | **Staff-Turnover-Ratio** | |
| --- | --- | --- | --- | --- | --- | --- | --- | --- |
|  | **Tolerance** | **VIF** | **Tolerance** | **VIF** | **Tolerance** | **VIF** | **Tolerance** | **VIF** |
| Year | 0.918 | 1.089 | 0.918 | 1.089 | 0.919 | 1.088 | 0.544 | 1.839 |
| Covid-19 dummy | 0.893 | 1.120 | 0.893 | 1.120 | 0.893 | 1.120 | 0.532 | 1.879 |
| High_Degree_Digitalization_HIMSS | 0.956 | 1.046 | 0.957 | 1.045 | 0.956 | 1.046 | 0.940 | 1.064 |
| Beds | 0.953 | 1.050 | 0.953 | 1.049 | 0.952 | 1.050 | 0.923 | 1.083 |
| Staff-to-Patient-Ratio | 0.890 | 1.123 | 0.890 | 1.124 | 0.891 | 1.122 | 0.962 | 1.040 |

| **Outcome Variable** | **Asset-Turnover-Ratio** | | **Number of Patient treated** | | **Length of Stay** | | **Absenteeism** | |
| --- | --- | --- | --- | --- | --- | --- | --- | --- |
|  | **Tolerance** | **VIF** | **Tolerance** | **VIF** | **Tolerance** | **VIF** | **Tolerance** | **VIF** |
| **Year** | 0.918 | 1.089 | 0.919 | 1.088 | 0.250 | 3.996 | 0.919 | 1.089 |
| **Covid-19 dummy** | 0.893 | 1.120 | 0.893 | 1.119 | 0.252 | 3.968 | 0.894 | 1.119 |
| **High_Degree_Digitalization_HIMSS** | 0.957 | 1.045 | 0.962 | 1.040 | 0.955 | 1.047 | 0.959 | 1.043 |
| **Beds** | 0.953 | 1.049 | 0.958 | 1.044 | 0.937 | 1.067 | 0.959 | 1.046 |
| **Staff-to-Patient-Ratio** | 0.890 | 1.124 | 0.891 | 1.122 | 0.943 | 1.060 | 0.890 | 1.124 |
