# Supplementary material for: Impact of Digitalization on Performance of Dutch Hospitals: Longitudinal Quantitative Study
Source: J Med Internet Res. 2026 Sep 29;28:e94896. doi: 10.2196/94896 (PMC13662437; doi:10.2196/94896)
Supplement: Supplementary file 7 [file jmir_v28i1e94896_app7.docx]

| **Table 1 – Hospitals with Newsweek’s Worlds SMARTEST Hospital Ranking** | | | |
| --- | --- | --- | --- |
| **Name** | ***Year*** | ***Rank*** | ***Source*** |
| **Erasmus Medische Centrum**^3^ | 2021 / 2023 | 57 / 50 | [World's Best Smart Hospitals 2021 - Newsweek Rankings](https://www.newsweek.com/worlds-best-smart-hospitals-2021)  [World's Best Smart Hospitals 2023 - Newsweek Rankings](https://www.newsweek.com/rankings/worlds-best-smart-hospitals-2023) |
| **Akademische Ziekenhuis Maastricht**^3^ | 2021 / 2023 | 83 / 89 | [World's Best Smart Hospitals 2021 - Newsweek Rankings](https://www.newsweek.com/worlds-best-smart-hospitals-2021)  [World's Best Smart Hospitals 2023 - Newsweek Rankings](https://www.newsweek.com/rankings/worlds-best-smart-hospitals-2023) |
| **Amsterdam UMC**^3^ | 2021 / 2023 | 100 / 97 | [World's Best Smart Hospitals 2021 - Newsweek Rankings](https://www.newsweek.com/worlds-best-smart-hospitals-2021)  [World's Best Smart Hospitals 2023 - Newsweek Rankings](https://www.newsweek.com/rankings/worlds-best-smart-hospitals-2023) |
| **UMC Utrecht** | 2021 / 2023 | 117 / 108 | [World's Best Smart Hospitals 2021 - Newsweek Rankings](https://www.newsweek.com/worlds-best-smart-hospitals-2021)  [World's Best Smart Hospitals 2023 - Newsweek Rankings](https://www.newsweek.com/rankings/worlds-best-smart-hospitals-2023) |
| **Jeoron Bosch Ziekenhuis** | 2021 / 2023 | 249 / 232 | [World's Best Smart Hospitals 2021 - Newsweek Rankings](https://www.newsweek.com/worlds-best-smart-hospitals-2021)  [World's Best Smart Hospitals 2023 - Newsweek Rankings](https://www.newsweek.com/rankings/worlds-best-smart-hospitals-2023) |
| **Radbound UMC** | 2023 | 251 | [World's Best Smart Hospitals 2023 - Newsweek Rankings](https://www.newsweek.com/rankings/worlds-best-smart-hospitals-2023) |
| **Catharina Ziekenhuis**^3^ | 2023 | 257 | [World's Best Smart Hospitals 2023 - Newsweek Rankings](https://www.newsweek.com/rankings/worlds-best-smart-hospitals-2023) |
| **BovenIJ Ziekenhuis**^3^ | 2023 | 284 | [World's Best Smart Hospitals 2023 - Newsweek Rankings](https://www.newsweek.com/rankings/worlds-best-smart-hospitals-2023) |

As there is no Dutch hospital, that fell out of the Top 350, these hospitals are used in the Mixed Models for 2022 as well.

| **Table 2 – Model descriptions** | |
| --- | --- |
| **Model** | **Description** |
| **Null Model** | Random intercept-only model (no predictors), used to estimate baseline ICC. |
| **Model 1** | Includes Year and Digitalization dummy |
| **Model 2** | Adds control variables: Teaching Status and Number of Beds to Model 1. |
| **Model 2.1** | Adds the Staff-to-Patient Ratio as a control variable to Model 2. |
| **Model 3** | Adds COVID-19 dummy to Model 2 (to control for pandemic effects). |
| **Model 3.1*** | Adds the Staff-to-Patient Ratio as a control variable to Model 3. |
| **Model 4** | Same as Model 2 but includes Year as a random effect. |
| **Model 4.1** | Adds Staff-to-Patient Ratio as a control variable to Model 4. |
| **Model 5** | Adds COVID-19 dummy to Model 4 (to control for pandemic effects). |
| **Model 5.1** | Adds Staff-to-Patient Ratio as a control variable to Model 4. |
| Note: * Reported Full Model in the manuscript | |

| **Table 3: Profit Margin – Newsweek's World Smartest Hospital Ranking Mixed-effect Model development** | | | | | |
| --- | --- | --- | --- | --- | --- |
| **Parameter** | **Null Model** | **Model 1** | **Model 2** | **Model 2.1** | **Model 3** |
| **Intercept** | 1.979 (1.184- 2.775)*** | -66.555 (-383.490- 244.381) | -88.253 (-406.067- 229.561) | -86.364 (-413.553- 240.825) | -120.696 (-438.167- 196.774) |
| ***Control Variables*** | | | | | |
| **Academic hospital** |  |  | -0.112 (-1.532- 1.307) | -0.026 (-1.473- 1.420) | -0.091 (-1.502- 1.321) |
| **Size (Number of Beds)** |  |  | 0.001 (-0.002- 0.003) | 0.001 (-0.002- 0.003) | 0.000 (-0.002- 0.003) |
| **Staff-to-Patient Ratio** |  |  |  | 1.988 (-3.708- 7.684) |  |
| ***Independent Variables*** | | | | | |
| **Year** |  | 0.035 (-0.120- 0.191) | 0.045 (-0.113- 0.202) | 0.044 (-0.118- 0.206) | 0.061 (-0.96- 0.218) |
| **COVID Year** |  |  |  |  | -0.460 (-0.819- -0.100)* |
| **Dummy high degree of digitalization** |  | -0.109 (-1.313- 1.094) | -0.121 (-1.368- 1.126) | -0.383 (-1.717- 0.952) | -0.095 (-1.333- 1.144) |
| **ICC** | 0.43 | 0.42 | 0.40 | 0.40 | 0.40 |
| **AIC** | 2176.386 | 2180.170 | 2113.433 | 2113.7491 | 2167.262 |
| **BIC** | 2193.172 | 2205.348 | 2150.807 | 2151.123 | 2204.975 |
| *Note:* Coefficients are reported with 95% confidence intervals in parentheses. * *p* < 0.05, ** p < 0.01, *** p < 0.001.  *Note:* Variance-Inflation Factors (VIF) are reported in Appendix F. All VIF values were below the threshold of 5 (mean = 1.8), indicating no relevant multicollinearity. | | | | |  |

| **Table 3: Profit Margin – Newsweek's World Smartest Hospital Ranking Mixed-effect Model development** | | | | | |
| --- | --- | --- | --- | --- | --- |
| **Parameter** | **Model 3.1** | **Model 4** | **Model 4.1** | **Model 5** | **Model 5.1** |
| **Intercept** | -118.742 (-445.935- 208.451) | -87.721 (-404.501- 229.059) | -85.741 (-411.814- 240.331) | -120.230 (-436.663- 196.203) | -118.173 (-444.234- 207.889) |
| ***Control Variables*** | | | | | |
| **Academic hospital** | -0.004 (-1.442- 1.434) | -0.133 (-1.532- 1.305) | -0.027 (-1.473- 1.419) | -0.092 (-1.502-1.319) | -0.005 (-1,443- 1.432) |
| **Size (Number of Beds)** | 0.000 (-0.002- 0.003) | 0.001 (-0.002- 0.003) | 0.001 (-0.002- 0.003) | 0.000 (-0.002- 0.003) | 0.000 (-0.002- 0.003) |
| **Staff-to-Patient Ratio** | 2.039 (-3.616- 7.694) |  | 1.990 (-3.706- 7.686) |  | 2.041 (-3.614- 7.695) |
| ***Independent Variables*** | | | | | |
| **Year** | 0.060 (-0.102- 0.222) | 0.044 (-0.113- 0.201) | 0.043 (0.118- 0.205) | 0.060 (-0.096- 0.217) | 0.056 (-0.102- 0.221) |
| **COVID Year** | -0.461 (-0.832- -0.090)* |  |  | -0.460 (-0.819- -0.100)* | -0.461 (-0.832- -0.089)* |
| **Dummy high degree of digitalization** | -0.350 (-1.675- 0.975) | -0.120 (-1.367- 1.126) | -0.382 (-1.716- 0.952) | -0.094 (-1.332- 1.144) | -0.349 (-1.674- 0.976) |
| **ICC** | 0.40 | 0.00 | 0.00 | 0.00 | 0.00 |
| **AIC** | 2109.517 | 2173.424 | 2115.321 | 2169.142 | 2111.407 |
| **BIC** | 2151.045 | 2211.137 | 2156.848 | 2211.046 | 2157.087 |
| *Note:* Coefficients are reported with 95% confidence intervals in parentheses. * *p* < 0.05, ** p < 0.01, *** p < 0.001.  *Note:* Variance-Inflation Factors (VIF) are reported in Appendix F. All VIF values were below the threshold of 5 (mean = 1.8), indicating no relevant multicollinearity. | | | | |  |

| **Table 4: Return on Assets (ROA) – Newsweek's World Smartest Hospital Ranking Mixed-effect Model development** | | | | | |
| --- | --- | --- | --- | --- | --- |
| **Parameter** | **Null Model** | **Model 1** | **Model 2** | **Model 2.1** | **Model 3** |
| **Intercept** | 0.159 (-0.113- 0.430) | -152.657 (-432.025-126.711) | -198.389 (-492.711- 95.934) | -220.943 (-526.131- 84.246 | -225.270 (-526.199- 75.658) |
| ***Control Variables*** | | | | | |
| **Academic hospital** |  |  | 1.743 (0.674- 2.813)** | 2.063 (0.913- 3.213)** | 1.814 (0.728- 2.900)** |
| **Size (Number of Beds)** |  |  | 0.000 (-0.001- 0.001) | 0.000 (-0.001- 0.001) | 0.000 (-0.001- 0.001) |
| **Staff-to-Patient Ratio** |  |  |  | -5.984 (-14.453- 2.485) |  |
| ***Independent Variables*** | | | | | |
| **Year** |  | 0.076 (-0.063- 0.214) | 0.098 (-0.047- 0.224) | 0.109 (-0.042- 0.261) | 0.112 (-0.037- 0.261) |
| **COVID Year** |  |  |  |  | -0.312 (-0.919- 0.295) |
| **Dummy high degree of digitalization** |  | -0.315 (-1.814- 1.184) | -1.229 (-2.874- 0.416) | -1.423 (-3.160- 0.314) | -1.266 (-2.921- 0.388) |
| **ICC** | 0.00 | 0.00 | 0.02 | 0.02 | 0.02 |
| **AIC** | 2490.092 | 2492.883 | 2474.002 | 2400.520 | 2475.011 |
| **BIC** | 2506.886 | 2518.074 | 2507.541 | 2437.914 | 2512.742 |
| *Note:* Coefficients are reported with 95% confidence intervals in parentheses. * *p* < 0.05, ** p < 0.01, *** p < 0.001.  *Note:* Variance-Inflation Factors (VIF) are reported in Appendix F. All VIF values were below the threshold of 5 (mean = 1.8), indicating no relevant multicollinearity. | | | | |  |

| **Table 4: Return on Assets – Newsweek's World Smartest Hospital Ranking Mixed-effect Model development** | | | | | |
| --- | --- | --- | --- | --- | --- |
| **Parameter** | **Model 3.1** | **Model 4** | **Model 4.1** | **Model 5** | **Model 5.1** |
| **Intercept** | -248.784 (-560.126- 62.559) | 1.862 (-1.337- 5.061) | 2.636 (-0.960- 6.232) | 1.422 (-2.015- 4.860) | 1.773 (-3.077- 6.624) |
| ***Control Variables*** | | | | | |
| **Academic hospital** | 2.129 (0.966- 3.292)*** | -0.003 (-0.015- 0.009) | -0.009 (-0.021- 0.004) | -0.003 (-0.016- 0.009) | -0.010 (-0.023- 0.003) |
| **Size (Number of Beds)** | -0.000 (-0.001- 0.001) | -0.000 (-0.000- 0.000) | -0.000 (-0.000- 0.000) | -0.000 (-0.000- 0.000) | -0.000 (-0.000- 0.000) |
| **Staff-to-Patient Ratio** | -6.001 (-14.479- 2.476) |  | 0.164 (0.059- 0.268)** |  | 0.160 (0.031- 0.289) |
| ***Independent Variables*** | | | | | |
| **Year** | 0.132 (-0.031- 0.277) | -0.001 (-0.002- 0.001) | -0.001 (-0.003- 0.000) | -0.001 (-0.002- 0.001) | -0.001 (-0.003- 0.002) |
| **COVID Year** | -0.328 (-0.956- 0.229) |  |  | -0.004 (-0.010- 0.002) | -0.005 (-0.023- 0.003) |
| **Dummy high degree of digitalization** | -1.456 (-3.202- 0.290) | 0.007 (-0.006- 0.021) | 0.005 (-0.009- 0.020) | 0.007 (-0.007- 0.022) | 0.001 (-0.009- 0.010) |
| **ICC** | 0.02 | 0.00 | 0.00 | 0.00 | 0.00 |
| **AIC** | 2401.485 | -1124.324 | -1333.347 | -1119.468 | -1344.136 |
| **BIC** | 2443.033 | -1082.503 | -1291.926 | -1077.668 | -1302.735 |
| *Note:* Coefficients are reported with 95% confidence intervals in parentheses. * *p* < 0.05, ** p < 0.01, *** p < 0.001.  *Note:* Variance-Inflation Factors (VIF) are reported in Appendix F. All VIF values were below the threshold of 5 (mean = 1.8), indicating no relevant multicollinearity. | | | | |  |

| **Table 5: Personnel-Expense-Ratio (PER) – Newsweek's World Smartest Hospital Ranking Mixed-effect Model development** | | | | | |
| --- | --- | --- | --- | --- | --- |
| **Parameter** | **Null Model** | **Model 1** | **Model 2** | **Model 2.1** | **Model 3** |
| **Intercept** | 48.909 (47.634- 50.185)*** | 1479.789 (776.745- 2182.832)*** | 1391.769 (700.037- 2083.501)*** | 1368.930 (666.086- 2071.773)*** | 1580.096 (915.424- 2244.767)*** |
| ***Control Variables*** | | | | | |
| **Academic hospital** |  |  | 4.489 (1.594-7.383)** | 4.468 (1.229- 7.108)** | 4.042 (1.258- 6.826)** |
| **Size (Number of Beds)** |  |  | 0.000 (-0.004- 0.004) | 0.000 (-0.004- 0.004) | 0.000 (-0.003- 0.005) |
| **Staff-to-Patient Ratio** |  |  |  | -0.277 (-13.065- 12.512) |  |
| ***Independent Variables*** | | | | | |
| **Year** |  | -0.708 (-1.056- 0.360)*** | -0.665 (-1.007- -0.322)*** | -0.654 (-1.002- -0.306)*** | -0.759 (-1.088- -0.430)*** |
| **COVID Year** |  |  |  |  | 3.040 (2.245- 3.834)*** |
| **Dummy high degree of digitalization** |  | 0.504 (-2.215- 3.223) | -0.790 (-3.624- 2.044) | -0.047 (-3.033- 2.938) | -0.828 (-3.494- 1.837) |
| **ICC** | 0.00 | 0.00 | 0.00 | 0.00 | 0.00 |
| **AIC** | 2926.169 | 2914.416 | 2892.710 | 2799.695 | 2841.907 |
| **BIC** | 2942.914 | 2939.533 | 2926.151 | 2836.992 | 2879.527 |
| *Note:* Coefficients are reported with 95% confidence intervals in parentheses. * *p* < 0.05, ** p < 0.01, *** p < 0.001.  *Note:* Variance-Inflation Factors (VIF) are reported in Appendix F. All VIF values were below the threshold of 5 (mean = 1.8), indicating no relevant multicollinearity. | | | | |  |

| **Table 5: Personnel-Expense-Ratio (PER) – Newsweek's World Smartest Hospital Ranking Mixed-effect Model development** | | | | | |
| --- | --- | --- | --- | --- | --- |
| **Parameter** | **Model 3.1** | **Model 4** | **Model 4.1** | **Model 5** | **Model 5.1** |
| **Intercept** | 1560.955 (888.171- 2233.739)*** | 1391.769 (700.037- 2083.501)*** | 1368.930 (666.086- 2071.773)*** | 1580.096 (915.424- 2244.767) | 1560.955 (888.171- 2233.739)*** |
| ***Control Variables*** | | | | | |
| **Academic hospital** | 3.681 (0.868- 6.495) | 4.489 (1.594- 7.383)*** | 4.168 (1.229- 7.108)** | 4.042 (1.258- 6.826)** | 3.681 (0.868-6.495)** |
| **Size (Number of Beds)** | 0.001 (-0.003- 0.005) | 0.000 (-0.004- 0.004) | 0.000 (-0.004- 0.004) | 0.000 (-0.003- 0.005) | 0.001 (-0.003- 0.005) |
| **Staff-to-Patient Ratio** | -1.148 (-13.079- 10.782) |  | -0.277 (-13.065- 12.512) |  | -1.148 (-13.079- 10.782) |
| ***Independent Variables*** | | | | | |
| **Year** | -0.749 (-1.082- -0.416)*** | -0.665 (-1.007- -0.322) | -0.654 (-1.002- 0.306)*** | -0.759 (-1.088- -0.430)*** | -0.749 (-1.082- -0.416)*** |
| **COVID Year** | 3.144 (2.342- 3.946)*** |  |  | 3.040 (2.245- 3.834)*** | 3.144 (2.342- 3.946)*** |
| **Dummy high degree of digitalization** | -0.091 (-2.882- 2.701) | -0.790 (-3.624- 2.044) | -0.047 (-3.033- 2.938) | -0.828 (-3.494- 1.837) | -0.091 (-2.882- 2.701) |
| **ICC** | 0.00 | 0.00 | 0.00 | 0.00 | 0.00 |
| **AIC** | 2746.721 | 2894.710 | 2801.6951 | 2843.907 | 2748.721 |
| **BIC** | 2788.163 | 2932.331 | 2843.136 | 2885.707 | 2794.307 |
| *Note:* Coefficients are reported with 95% confidence intervals in parentheses. * *p* < 0.05, ** p < 0.01, *** p < 0.001.  *Note:* Variance-Inflation Factors (VIF) are reported in Appendix F. All VIF values were below the threshold of 5 (mean = 1.8), indicating no relevant multicollinearity. | | | | |  |

| **Table 6: Asset-Turnover-Ratio – Newsweek's World Smartest Hospital Ranking Mixed-effect Model development** | | | | | |
| --- | --- | --- | --- | --- | --- |
| **Parameter** | **Null Model** | **Model 1** | **Model 2** | **Model 2.1** | **Model 3** |
| **Intercept** | 0.053 (0.027- 0.079)*** | 14.710 (-12.328- 41.748) | 14..856 (-12.438- 42.150) | 14.434 (10.842- 18.026)*** | 18.204 (-9.471- 45.878) |
| ***Control Variables*** | | | | | |
| **Academic hospital** |  |  | 0.029 (-0.065- 0.123) | 0.019 (0.005- 0.032)** | 0.028 (-0.066- 0.122) |
| **Size (Number of Beds)** |  |  | 0.000 (-0.000- 0.000) | 0.000 (-0.000- 0.000) | 0.000 (-0.000- 0.000) |
| **Staff-to-Patient Ratio** |  |  |  | 0.005 (-0.079- 0.090) |  |
| ***Independent Variables*** | | | | | |
| **Year** |  | -0.007 (-0.021- 0.006) | -0.007 (-0.021- 0.006) | -0.007 (-0.009- -0.005)*** | -0.009 (-0.023- 0.005) |
| **COVID Year** |  |  |  |  | 0.040 (-0.018- 0.099) |
| **Dummy high degree of digitalization** |  | -0.008 (-0.153- 0.138) | -0.009 (-0.164- 0.146) | -0.007 (-0.025- 0.012) | -0.006 (-0.161- 0.149) |
| **ICC** | 0.00 | 0.00 | 0.00 | 0.00 | 0.00 |
| **AIC** | 189.780 | 200.531 | 205.451 | -1972.074 | 205.636 |
| **BIC** | 214.566 | 225.710 | 238.973 | -1916.680 | 243.349 |
| *Note:* Coefficients are reported with 95% confidence intervals in parentheses. * *p* < 0.05, ** p < 0.01, *** p < 0.001.  *Note:* Variance-Inflation Factors (VIF) are reported in Appendix F. All VIF values were below the threshold of 5 (mean = 1.8), indicating no relevant multicollinearity. | | | | |  |

| **Table 6: Asset-Turnover-Ratio (ATR) – Newsweek's World Smartest Hospital Ranking Mixed-effect Model development** | | | | | |
| --- | --- | --- | --- | --- | --- |
| **Parameter** | **Model 3.1** | **Model 4** | **Model 4.1** | **Model 5** | **Model 5.1** |
| **Intercept** | 14.367 (10.762- 17.973)*** | 14.856 (-12.438- 42.150) | 14.434 (10.842- 18.026)*** | 18.204 (-9.471- 45.878) | 14.367 (10.762- 17.976)*** |
| ***Control Variables*** | | | | | |
| **Academic hospital** | 0.019 (0.005- 0.032)** | 0.029 (-0.065- 0.123) | 0.019 (0.005- 0.032) | 0.028 (-0.066- 0.122) | 0.019 (0.005- 0.032)** |
| **Size (Number of Beds)** | -0.000 (-0.000- 0.000) | -0.000 (-0.000- 0.000) | -0.000 (-0.000- 0.000) | -0.000 (-0.000- 0.000) | 0.000 (-0.000- 0.000) |
| **Staff-to-Patient Ratio** | 0.006 (-0.079- 0.090) |  | 0.005 (-0.079- 0.090) |  | 0.006 (-0.079- 0.090) |
| ***Independent Variables*** | | | | | |
| **Year** | -0.007 (-0.009- -0.005)*** | -0.007 (-0.021- 0.006) | -0.007 (-0.009- -0.005)*** | -0.009 (-0.023- 0.005) | -0.007 (-0.009- -0.005)*** |
| **COVID Year** | -0.001 (-0.007- 0.005) |  |  | 0.040 (-0.018- 0.099) | -0.001 (-0.079- 0.005) |
| **Dummy high degree of digitalization** | -0.007 (-0.025- 0.012) | -0.009 (-0.164- 0.146) | -0.007 (-0.025- 0.012) | -0.006 (-0.161- 0.149) | -0.007 (-0.02- 0.012) |
| **ICC** | 0.00 | 0.00 | 0.00 | 0.00 | 0.00 |
| **AIC** | -1952.204 | 207.451 | -1952.074 | 207.636 | -1950.204 |
| **BIC** | -1910.655 | 245.134 | -1910.525 | 249.539 | -1904.500 |
| *Note:* Coefficients are reported with 95% confidence intervals in parentheses. * *p* < 0.05, ** p < 0.01, *** p < 0.001.  *Note:* Variance-Inflation Factors (VIF) are reported in Appendix F. All VIF values were below the threshold of 5 (mean = 1.8), indicating no relevant multicollinearity. | | | | |  |

| **Table 7: Number of Patients Treated – Newsweek's World Smartest Hospital Ranking Mixed-effect Model development** | | | | | |
| --- | --- | --- | --- | --- | --- |
| **Parameter** | **Null Model** | **Model 1** | **Model 2** | **Model 2.1** | **Model 3** |
| **Intercept** | 137317.969 (120441.864- 154194.074)*** | 135212.248 (118685.165- 151739.331)*** | 30276.599 (10219.539- 50333.659)*** | 53809.264 (33396.636- 74221.892)*** | 35513.039 (15039.842- 55986.235)*** |
| ***Control Variables*** | | | | | |
| **Academic hospital** |  |  | -52099.557 (-84405.932- -19793.181)** | -33587.847(-65339.822- -1835.871)* | -52155.795 (-84228.917- -200082.673)** |
| **Size (Number of Beds)** |  |  | 211.474 (174.844- 248.103)*** | 208.452 (173.500- 243.405)*** | 209.598 (173.165- 246.030)*** |
| **Staff-to-Patient Ratio** |  |  |  | -663464.264 (-867531.195- -459397.333)*** |  |
| ***Independent Variables*** | | | | | |
| **Year** |  | 0.001 (.) | 0.001 (.) | 0.002 (.) | 0.002 (.) |
| **COVID Year** |  |  |  |  | -16815.709 (-32102.082- -1529.335)* |
| **Dummy high degree of digitalization** |  | 44377.945 (-3354.584- 92110.474) | 45689.424 (526.867- 90851.980)* | 43497.927 (-213.806- 87209.660) | 47389.084 (2562.619- 92215.548)* |
| **ICC** | 0.27 | 0.20 | 0.00 | 0.00 | 0.00 |
| **AIC** | 12249.054 | 12250.002 | 12108.101 | 11949.448 | 12105.474 |
| **BIC** | 12265.758 | 12275.057 | 12141.458 | 11986.880 | 12143.000 |
| *Note:* Coefficients are reported with 95% confidence intervals in parentheses. * *p* < 0.05, ** p < 0.01, *** p < 0.001.  *Note:* Variance-Inflation Factors (VIF) are reported in Appendix F. All VIF values were below the threshold of 5 (mean = 1.8), indicating no relevant multicollinearity. | | | | |  |

| **Table 7: Number of Patients Treated – Newsweek's World Smartest Hospital Ranking Mixed-effect Model development** | | | | | | | |
| --- | --- | --- | --- | --- | --- | --- | --- |
| **Parameter** | **Model 3.1** | **Model 4** | **Model 4.1** | **Model 5** | **Model 5.1** | | |
| **Intercept** | 58379.364 (37616.941- 79141.787)*** | 30289.190 (10221.057- 50357.323)*** | 53817.082 (33396.608- 74237.556)*** | 35528.725 (15044.773- 56012.677)*** | | 58389.299 (37619.147- 79159.450)*** | |
| ***Control Variables*** | | | | | | | |
| **Academic hospital** | -33783.387 (-65340.782- -2225.992)* | -52058.514 (-84371.557- -19745.471)** | -33560.032 (-65316.296- -1803.768)* | -52114.895 (-84194.119- -20035.671)** | | | -33755.780 (-65317.316- -2194.245)* |
| **Size (Number of Beds)** | 206.871 (172.084- 241.659)*** | 211.434 (174.787- 248.081)*** | 208.421 (173.455- 243.387)*** | 209.555 (173.105- 246.004) | | | 206.838 (172.037- 241.639)*** |
| **Staff-to-Patient Ratio** | -656708.204 (-860029.197- -453387.211)*** |  | -663409.389 (-867488.462- -459330.315)*** |  | | | -656641.565 (859974.933- -453308.197)*** |
| ***Independent Variables*** | | | | | | | |
| **Year** | 0.002 (.) | 0.001 (.) | 0.002 (.) | 0.002 (.) | | | 0.002 (.) |
| **COVID Year** | -15520.885 (-30388.217- -653.553)* |  |  | -16819.904 (-32102.421- -1537.387) | | | -15523.608 (-30387.831- -659.386)* |
| **Dummy high degree of digitalization** | 44878.306 (1435.193- 88321.418)* | 45721.390 (565.389- -90877.390)* | 43536.284 (-168.866- 87241.434) | 47422.666 (2603.001- 92242.332)* | | | 44917.317 (1480.487- 88354.146)* |
| **ICC** | 0.00 | 0.00 | 0.00 | 0.00 | | | 0.00 |
| **AIC** | 11947.268 | 12110.082 | 11951.440 | 12107.450 | | | 11949.257 |
| **BIC** | 11988.859 | 12147.608 | 11993.031 | 12149.146 | | | 11995.007 |
| *Note:* Coefficients are reported with 95% confidence intervals in parentheses. * *p* < 0.05, ** p < 0.01, *** p < 0.001.  *Note:* Variance-Inflation Factors (VIF) are reported in Appendix F. All VIF values were below the threshold of 5 (mean = 1.8), indicating no relevant multicollinearity. | | | | | | |  |

| **Table 8: Length of Stay (LOS) – Newsweek's World Smartest Hospital Ranking Mixed-effect Model development** | | | | | |
| --- | --- | --- | --- | --- | --- |
| **Parameter** | **Null Model** | **Model 1** | **Model 2** | **Model 2.1** | **Model 3** |
| **Intercept** | 1.159 (0.915- 1.404)*** | 69.616 (42.919- 96.313)*** | 70.295 (42.033- 98.557)*** | 154.965 (119.117- 190.813)*** | 37.591 (-33.862- 109.045) |
| ***Control Variables*** | | | | | |
| **Academic hospital** |  |  | -0.029 (-0.439- 0.381) | -0.157 (-0.549- 0.236) | -0.037 (-0.446- 0.373) |
| **Size (Number of Beds)** |  |  | 0.000 (-0.000- 0.000) | 0.000 (-0.000- 0.000) | 0.000 (-0.000- 0.000) |
| **Staff-to-Patient Ratio** |  |  |  | 15.603 (13.740- 17.466)*** |  |
| ***Independent Variables*** | | | | | |
| **Year** |  | -0.034 (-0.047- -0.021)*** | -0.034 (-0.048- -0.020)*** | -0.076 (-0.094- -0.059)*** | -0.018 (-0.053- 0.017) |
| **COVID Year** |  |  |  |  | -0.049 (-0.148- 0.050) |
| **Dummy high degree of digitalization** |  | -0.118 (-0.341- 0.105) | -0.117 (-0.340- 0.106) | -0.138 (-0.347- 0.070) | -0.107 (-0.330- 0.117) |
| **ICC** | 0.92 | 0.93 | 0.93 | 0.93 | 0.93 |
| **AIC** | 392.906 | 369.270 | 373.234 | 244.629 | 374.271 |
| **BIC** | 408.222 | 392.233 | 403.865 | 278.983 | 408.732 |
| *Note:* Coefficients are reported with 95% confidence intervals in parentheses. * *p* < 0.05, ** p < 0.01, *** p < 0.001.  *Note:* Variance-Inflation Factors (VIF) are reported in Appendix F. All VIF values were below the threshold of 5 (mean = 1.8), indicating no relevant multicollinearity. | | | | |  |

| **Table 8: Length of Stay (LOS) – Newsweek's World Smartest Hospital Ranking Mixed-effect Model development** | | | | | |
| --- | --- | --- | --- | --- | --- |
| **Parameter** | **Model 3.1** | **Model 4** | **Model 4.1** | **Model 5** | **Model 5.1** |
| **Intercept** | 109.224 (44.796- 173.652)*** | 70.331 (42.050- 98.611)*** | 155.023 (119.156- 190.889)*** | 37.617 (-33.887- 109.120) | 109.197 (44.782- 173.612)*** |
| ***Control Variables*** | | | | | |
| **Academic hospital** | -0.155 (-0.548- 0.238) | -0.031 (-0.441- 0.379) | -0.157 (-0.549- 0.235) | -0.039 (-0.448- 0.371) | -0.155 (-0.548- 0.238) |
| **Size (Number of Beds)** | 0.000 (-0.000- 0.000) | 0.000 (-0.000- 0.000) | 0.000 (-0.000- 0.000) | 0.000 (-0.000- 0.000) | 0.000 (-0.000- 0.000) |
| **Staff-to-Patient Ratio** | 15.754 (13.913- 17.594)*** |  | 15.613 (13.750- 17.477)*** |  | 15.764 (13.923- 17.604)*** |
| ***Independent Variables*** | | | | | |
| **Year** | -0.054 (-0.086- -0.022) | -0.034 (-0.048- -0.020)*** | -0.076 (-0.094- -0.059)*** | -0.018 (-0.053- 0.017) | -0.054 (-0.086- -0.022)** |
| **COVID Year** | -0.078 (-0.168- 0.012) |  |  | -0.049 (-0.148- 0.050) | -0.078 (-0.168- 0.012) |
| **Dummy high degree of digitalization** | -0.141 (-0.350- 0.068) | -0.117 (-0.341- 0.106) | -0.138 (-0.094- -0.059) | -0.107 (-0.331- 0.117) | -0.141 (-0.350- 0.068) |
| **ICC** | 0.96 | 0.00 | 0.00 | 0.00 | 0.00 |
| **AIC** | 243.961 | 375.469 | 246.599 | 376.508 | 245.918 |
| **BIC** | 282.132 | 409.930 | 284.770 | 414.797 | 287.906 |
| *Note:* Coefficients are reported with 95% confidence intervals in parentheses. * *p* < 0.05, ** p < 0.01, *** p < 0.001.  *Note:* Variance-Inflation Factors (VIF) are reported in Appendix F. All VIF values were below the threshold of 5 (mean = 1.8), indicating no relevant multicollinearity. | | | | |  |

| **Table 9: Absenteeism – Newsweek's World Smartest Hospital Ranking Mixed-effect Model development** | | | | | |
| --- | --- | --- | --- | --- | --- |
| **Parameter** | **Null Model** | **Model 1** | **Model 2** | **Model 2.1** | **Model 3** |
| **Intercept** | 5.785 (5.565- 6.004)*** | -695.020 (-779.637- -610.402)*** | -697.290 (-782.475- -612.105)*** | -682.327 (-774.613- -590.042) | -699.219 (-786.773- -611.664)*** |
| ***Control Variables*** | | | | | |
| **Academic hospital** |  |  | -0.140 (-0.602- 0.323) | -0.226 (-0.752- 0.220) | -0.141 (-0.603- 0.322) |
| **Size (Number of Beds)** |  |  | 0.000 (-0.001- 0.001) | 0.000 (-0.001- 0.001) | 0.000 (-0.001- 0.001) |
| **Staff-to-Patient Ratio** |  |  |  | 0.347 (-2.440- 3.135) |  |
| ***Independent Variables*** | | | | | |
| **Year** |  | 0.347 (0.305- 0.389) | 0.348 (0.306- 0.390)*** | 0.341 (0.295- 0.386)*** | 0.349 (0.306- 0.392)*** |
| **COVID Year** |  |  |  |  | -0.020 (-0194- 0.155) |
| **Dummy high degree of digitalization** |  | -0.280 (-0.801- 0.242) | -0.253 (-0.780- 0.274) | -0.133 (-0.697- 0.431) | -0.255 (-0.783- 0.273) |
| **ICC** | 0.12 | 0.49 | 0.48 | 0.44 | 0.48 |
| **AIC** | 1528.588 | 1403.687 | 1396.215 | 1353.893 | 1398.167 |
| **BIC** | 1545.390 | 1428.890 | 1429.770 | 1391.306 | 1435.917 |
| *Note:* Coefficients are reported with 95% confidence intervals in parentheses. * *p* < 0.05, ** p < 0.01, *** p < 0.001.  *Note:* Variance-Inflation Factors (VIF) are reported in Appendix F. All VIF values were below the threshold of 5 (mean = 1.8), indicating no relevant multicollinearity. | | | | |  |

| **Table 9: Absenteeism – Newsweek's World Smartest Hospital Ranking Mixed-effect Model development** | | | | | |
| --- | --- | --- | --- | --- | --- |
| **Parameter** | **Model 3.1** | **Model 4** | **Model 4.1** | **Model 5** | **Model 5.1** |
| **Intercept** | -687.919 (-782.701- -593.137)*** | -697.387 (-782.502- -612.273)*** | -682.420 (-774.628- -590.212)*** | -699.300 (-786.786- -611.814)*** | -688.000 (-782.705- -593.295)*** |
| ***Control Variables*** | | | | | |
| **Academic hospital** | -0.269 (-0.756- 0.217) | -0.139 (-0.601- 0.323) | -0.265 (-0.751- 0.220) | -0.140 (-0.603- 0.322) | -0.269 (-0.756- 0.218) |
| **Size (Number of Beds)** | 0.000 (-0.001- 0.001) | 0.000 (-0.001- 0.001) | 0.000 (-0.001- 0.001) | 0.000 (-0.001- 0.001) | 0.000 (-0.001- 0.001) |
| **Staff-to-Patient Ratio** | 0.341 (-2.447- 3.129) |  | 0.348 (-2.440- 3.135) |  | 0.342 (-2.446- 3.129) |
| ***Independent Variables*** | | | | | |
| **Year** | 0.343 (0.297- 0.390) | 0.348 (0.306- 0.390)*** | 0.341 (0.295- 0.386)*** | 0.349 (0.306- 0.392)*** | 0.344 (0.297- 0.390)*** |
| **COVID Year** | -0.066 (-0.250- 0.118) |  |  | -0.140 (-0.603- 0.322) | -0.065 (-0.249- 0.119) |
| **Dummy high degree of digitalization** | -0.137 (-0.703- 0.429) | -0.252 (-0.780- 0.275) | -0.133 (-0.697- 0.431) | -0.254 (-0.782- 0.273) | -0.136 (-0.702- 0.430) |
| **ICC** | 0.44 | 0.00 | 0.00 | 0.00 | 0.00 |
| **AIC** | 1355.417 | 1398.023 | 1355.724 | 1399.977 | 1357.251 |
| **BIC** | 1396.987 | 1435.773 | 1397.293 | 1441.921 | 1402.978 |
| *Note:* Coefficients are reported with 95% confidence intervals in parentheses. * *p* < 0.05, ** p < 0.01, *** p < 0.001.  *Note:* Variance-Inflation Factors (VIF) are reported in Appendix F. All VIF values were below the threshold of 5 (mean = 1.8), indicating no relevant multicollinearity. | | | | |  |
