# Supplementary material for: Impact of Digitalization on Performance of Dutch Hospitals: Longitudinal Quantitative Study
Source: J Med Internet Res. 2026 Sep 29;28:e94896. doi: 10.2196/94896 (PMC13662437; doi:10.2196/94896)
Supplement: Supplementary file 8 [file jmir_v28i1e94896_app8.docx]

**Table 1 Power Analysis**

| **Assumed effect of digital maturity** | **Nested model R²** | **Full model R²** | **ΔR²** | **Full model predictors** | **Nested model predictors** | **Target power** | **α** | **Required N** |
| --- | --- | --- | --- | --- | --- | --- | --- | --- |
| Very Small | 0.10 | 0.11 | 0.01 | 11 | 10 | 0.80 | 0.05 | 1455 |
| Small | 0.10 | 0.12 | 0.02 | 11 | 10 | 0.80 | 0.05 | 724 |
| Moderate | 0.10 | 0.15 | 0.05 | 11 | 10 | 0.80 | 0.05 | 286 |
| Table Note: The analysis was conducted in IBM SPSS Statistics using the univariate linear regression power procedure. The nested model represents the covariate-only model, whereas the full model adds digital maturity as the predictor of interest. Required sample sizes refer to hospital-year observations. Because the primary analyses used linear mixed-effects models with repeated observations nested within hospitals, these estimates should be interpreted as approximate sensitivity analyses rather than exact power calculations for the mixed-effects models. | | | | | | | | |
