# Supplementary material for: Impact of Digitalization on Performance of Dutch Hospitals: Longitudinal Quantitative Study
Source: J Med Internet Res. 2026 Sep 29;28:e94896. doi: 10.2196/94896 (PMC13662437; doi:10.2196/94896)
Supplement: Supplementary file 9 [file jmir_v28i1e94896_app9.docx]

| **Table 1 – Hospitals with HIMSS Stages 6 or 7 in the Netherlands** | | | | |
| --- | --- | --- | --- | --- |
| **Name** | **First public Stage 6 / year** | **Upgrade / Reclassification year (if applicable)** | **Exposure Status at baseline** | **Source** |
| **Spaarne Ziekenhuis / Spaare Gasthuis** | 2011 | - | Advanced before baseline | [Spaarne Ziekenhuis ontvangt onderscheiding van HIMSS Analytics Europe voor inzet EPD - MedicalFacts.nl](https://www.medicalfacts.nl/2011/07/08/spaarne-ziekenhuis-ontvangt-onderscheiding-van-himss-analytics-europe-voor-inzet-epd/) |
| **Westfiesgasthuis (Hoorn) ^1^** | 2012 | - | Advanced before baseline | [Westfriesgasthuis behaalt prestigieuze onderscheiding voor verregaande automatisering patiÃ«ntendossiers - MedicalFacts.nl](https://www.medicalfacts.nl/2012/10/12/westfriesgasthuis-behaalt-prestigieuze-onderscheiding-voor-verregaande-automatisering-patientendossiers/) |
| **Sint Lucas Andreas Ziekenhuis^2^** | 2012 | 2018 | Change within the baseline | [Sint Lucas Andreas Ziekenhuis krijgt EPD-award - Computable.nl](https://www.computable.nl/2012/11/14/sint-lucas-andreas-ziekenhuis-krijgt-epd-award/)  [HIMSS22 Europe Celebrates Healthcare Systems Validated at EMRAM Stages 6 and 7 \| HIMSS](https://iowa.himss.org/news/himss22-europe-celebrates-healthcare-systems-validated-emram-stages-6-and-7) |
| **Amphia Ziekenhuis** | 2013 | - | Advanced before baseline | [Amphia bereikt HIMSS stage 6 met elektronisch patiëntendossier (EPD) - MedicalFacts.nl](https://www.medicalfacts.nl/2013/11/14/198229/) |
| **Leiden University Medical Center** | 2013 | - | Advanced before baseline | [LUMC krijgt eveneens prestigieus ICT-certificaat - Skipr](https://www.skipr.nl/nieuws/lumc-krijgt-eveneens-prestigieus-ict-certificaat/) |
| **University Medical Center Utrecht** | 2013 | - | Advanced before baseline | [UMC Utrecht verdient prestigieus ICT-certificaat - Skipr](https://www.skipr.nl/nieuws/umc-utrecht-verdient-prestigieus-ict-certificaat/) |
| **Ziekenhuisgroep Twente – ZGT** | 2014 | - | Advanced before baseline | [Hoge onderscheiding voor ZGT - MedicalFacts.nl](https://www.medicalfacts.nl/2014/03/18/hoge-onderscheiding-voor-zgt/) |
| **Maasstad Ziekenhuis** | 2014 | - | Advanced before baseline | [Maasstad Ziekenhuis ontvangt award voor elektronisch patiëntendossier - MedicalFacts.nl](https://www.medicalfacts.nl/2014/04/03/maasstad-ziekenhuis-ontvangt-award-voor-elektronisch-patientendossier/) |
| **Zaans Medisch Centrum** | 2014 | - | Advanced before baseline | [epd Archieven - Page 39 of 126 - Zorgvisie](https://www.zorgvisie.nl/tag/epd/page/39/) |
| **Jeroen Bosch Ziekenhuis** | 2015 | - | Advanced before baseline | ['Bij het EPD is de balans tussen privacy en medische efficiëntie doorgeschoten' - IT Pro - Nieuws - Tweakers](https://tweakers.net/nieuws/121403/bij-het-epd-is-de-balans-tussen-privacy-en-medische-efficientie-doorgeschoten.html) |
| **Radboud University Medical Center** | 2015 | 2017 (Stage 7) | Change within baseline | [Radboud University Medical Center Becomes First Hospital in the Netherlands to Win HIMSS Stage 7 Award](https://www.digitalhealthnews.eu/industry/4253-radboud-university-medical-center-becomes-first-hospital-in-the-netherlands-to-win-himss-stage-7-award)  [2017 EMRAM trailblazers \| Healthcare IT News](https://www.healthcareitnews.com/news/2017-emram-trailblazers) |
| **Stichting Antonius Ziekenhuis** | 2016 | - | Advanced before baseline | [HIMSS EMRAM Stage 6 & 7 Community \| HIMSS Europe](https://web.archive.org/web/20161210030819/http:/www.himss.eu/communities/himss-emram-stage-6-7-community) |
| **St. Jansdal Ziekenhuis** | 2016 | 2017 (Stage 7) | Change within the baseline | [‘Opereer agile en excelleer aan de zachte kant’ \| ICT&health](https://icthealth.nl/magazine/editie-02-2017/opereer-agile-en-excelleer-aan-de-zachte-kant) |

^1^ Merged to Dijklander Ziekenhuis

^2^ Merged to OLVG

| **Table 2 – Model descriptions** | |
| --- | --- |
| **Model** | **Description** |
| **Null Model** | Random intercept-only model (no predictors), used to estimate baseline ICC. |
| **Model 1** | Includes Year and Digitalization dummy |
| **Model 2** | Adds control variables: Teaching Status and Number of Beds to Model 1. |
| **Model 2.1** | Adds the Staff-to-Patient Ratio as a control variable to Model 2. |
| **Model 3** | Adds COVID-19 dummy to Model 2 (to control for pandemic effects). |
| **Model 3.1*** | Adds the Staff-to-Patient Ratio as a control variable to Model 3. |
| **Model 4** | Same as Model 2 but includes Year as a random effect. |
| **Model 4.1** | Adds Staff-to-Patient Ratio as a control variable to Model 4. |
| **Model 5** | Adds COVID-19 dummy to Model 4 (to control for pandemic effects). |
| **Model 5.1** | Adds Staff-to-Patient Ratio as a control variable to Model 4. |
| Note: * Reported Full Model in the manuscript | |

| **Table 3: Profit Margin – HIMSS EMRAM excluding hospitals that were reclassified or upgraded to HIMSS Stage 6 or 7 during the study period Mixed-effect Model development** | | | | | |
| --- | --- | --- | --- | --- | --- |
| **Parameter** | **Null Model** | **Model 1** | **Model 2** | **Model 2.1** | **Model 3** |
| **Intercept** | 2.02 (1.196- 2.843)*** | -71.238 (-394.085- 251.608) | -90.706 (-417.565- 236.152) | -68.964 (-403.699- 265.771) | -123.186 (-449.857- 203.485) |
| ***Control Variables*** | | | | | |
| **Academic hospital** |  |  | -0.012 (-1.645- 1.620) | -0.001 (-1.654- 1.653) | 0.032 (-1.592- 1.656) |
| **Size (Number of Beds)** |  |  | 0.001 (-0.002- 0.003) | 0.001 (-0.002- 0.003) | 0.000 (-0.002- 0.003) |
| **Staff-to-Patient Ratio** |  |  |  | 1.908 (-3.879- 7.694) |  |
| ***Independent Variables*** | | | | | |
| **Year** |  | 0.036 (-0.124- 0.196) | 0.046 (-0.116- 0.208) | 0.035 (-0.131- 0.201) | 0.062 (-0.100- 0.224) |
| **COVID Year** |  |  |  |  | -0.450 (-0.822- -0.078)* |
| **Dummy high degree of digitalization** |  | -0.004 (-2.039- 2.031) | -0.070 (-2.114- 1.974) | -0.072 (-2.132- 1.988) | -0.054 (-2.092- 1.983) |
| **ICC** | 0.42 | 0.41 | 0.39 | 0.39 | 0.38 |
| **AIC** | 2111.058 | 2114.855 | 2106.495 | 2047.019 | 2102.555 |
| **BIC** | 2127.695 | 2139.809 | 2139.405 | 2084.042 | 2139.930 |
| *Note:* Coefficients are reported with 95% confidence intervals in parentheses. * *p* < 0.05, ** p < 0.01, *** p < 0.001.  *Note:* Variance-Inflation Factors (VIF) are reported in Appendix F. All VIF values were below the threshold of 5 (mean = 1.8), indicating no relevant multicollinearity. | | | | |  |

| **Table 3: Profit Margin – HIMSS EMRAM excluding hospitals that were reclassified or upgraded to HIMSS Stage 6 or 7 during the study period Mixed-effect Model development** | | | | | |
| --- | --- | --- | --- | --- | --- |
| **Parameter** | **Model 3.1** | **Model 4** | **Model 4.1** | **Model 5** | **Model 5.1** |
| **Intercept** | -102.949 (-438.098- 232.199) | -90.076 (-415.760- 235.608) | -68.279 (-401.834- 265.277) | -122.614 (-448.107- 202.880) | -102.302 (-436.241- 231.637) |
| ***Control Variables*** | | | | | |
| **Academic hospital** | 0.049 (-1.596- 1.694) | -0.014 (-1.646- 1.618) | -0.001 (-1.654- 1.651) | 0.030 (-1.594- 1.654) | 0.048 (-1.596- 1.692) |
| **Size (Number of Beds)** | 0.000 (-0.002- 0.003) | 0.001 (-0.002- 0.003) | 0.001 (-0.002- 0.003) | 0.000 (-0.002- 0.003) | 0.000 (-0.002- 0.003) |
| **Staff-to-Patient Ratio** | 1.949 (-3.795- 7.694) |  | 1.910 (-3.877- 7.696) |  | 1.951 (-3.793- 7.695) |
| ***Independent Variables*** | | | | | |
| **Year** | 0.052 (-0.114- 0.218) | 0.045 (-0.116- 0.207) | 0.035 (-0.131- 0.200) | 0.062 (-0.099- 0.223) | 0.052 (-0.114- 0.217) |
| **COVID Year** | -0.466 (-0.850- -0.082)* |  |  | -0.450 (-0.822- -0.078)* | -0.466 (-0.850- -0.082) |
| **Dummy high degree of digitalization** | -0.051 (-2.104- 2.003) | -0.071 (-2.116- 1.975) | -0.073 (-2.134- 1.989) | -0.055 (-2.094- 1.984) | -0.051 (-2.105- 2.003) |
| **ICC** | 0.38 | 0.00 | 0.00 | 0.00 | 0.00 |
| **AIC** | 2043.259 | 2108.069 | 2048.906 | 2104.442 | 2045.248 |
| **BIC** | 2084.495 | 2145.444 | 2090.043 | 2145.970 | 2090.499 |
| *Note:* Coefficients are reported with 95% confidence intervals in parentheses. * *p* < 0.05, ** p < 0.01, *** p < 0.001.  *Note:* Variance-Inflation Factors (VIF) are reported in Appendix F. All VIF values were below the threshold of 5 (mean = 1.8), indicating no relevant multicollinearity. | | | | |  |

| **Table 4: Return on Assets (ROA) – HIMSS EMRAM excluding hospitals that were reclassified or upgraded to HIMSS Stage 6 or 7 during the study period Mixed-effect Model development** | | | | | |
| --- | --- | --- | --- | --- | --- |
| **Parameter** | **Null Model** | **Model 1** | **Model 2** | **Model 2.1** | **Model 3** |
| **Intercept** | 0.164 (-0.118- 0.446) | -148.295 (-431.919- 135.328) | -154.353 (-453.454- 144.748) | -172.808 (-484.185- 138.569) | -181.681 (-488.198- 124.837) |
| ***Control Variables*** | | | | | |
| **Academic hospital** |  |  | 1.795 (0.656- 2.935)** | 2.088 (0.874- 3.302)** | 1.890 (0.728- 3.051)** |
| **Size (Number of Beds)** |  |  | 0.000 (-0.001- 0.001) | 0.000 (-0.002- 0.001) | 0.000 (-0.002- 0.001) |
| **Staff-to-Patient Ratio** |  |  |  | -6.026 (-14.703- 2.651) |  |
| ***Independent Variables*** | | | | | |
| **Year** |  | 0.074 (-0.067- 0.214) | 0.076 (-0.072- 0.225) | 0.086 (-0.068- 0.240) | 0.090 (-0.062- 0.242) |
| **COVID Year** |  |  |  |  | -0.327 (-0.958- 0.305) |
| **Dummy high degree of digitalization** |  | -0.182 (-0.990- 0.627) | -0.319 (-1.265- 0.627) | -0.342 (-1.321- 0.638) | -0.323 (-1.291- 0.646) |
| **ICC** | 0.00 | 0.00 | 0.00 | 0.02 | 0.02 |
| **AIC** | 2416.907 | 2419.653 | 2401.588 | 2327.707 | 2402.978 |
| **BIC** | 2433.552 | 2444.620 | 2434.827 | 2364.751 | 2439.982 |
| *Note:* Coefficients are reported with 95% confidence intervals in parentheses. * *p* < 0.05, ** p < 0.01, *** p < 0.001.  *Note:* Variance-Inflation Factors (VIF) are reported in Appendix F. All VIF values were below the threshold of 5 (mean = 1.8), indicating no relevant multicollinearity. | | | | |  |

| **Table 4: Return on Assets – HIMSS EMRAM excluding hospitals that were reclassified or upgraded to HIMSS Stage 6 or 7 during the study period Mixed-effect Model development** | | | | | |
| --- | --- | --- | --- | --- | --- |
| **Parameter** | **Model 3.1** | **Model 4** | **Model 4.1** | **Model 5** | **Model 5.1** |
| **Intercept** | -201.716 (-520.102- 116.670) | -154.439 (-453.483- 144.605) | -172.900 (-484.205- 138.405) | -181.805 (-488.265- 124.656) | -201.852 (-520.173- 116.470) |
| ***Control Variables*** | | | | | |
| **Academic hospital** | 2.179 (0.947- 3.411)*** | 1.800 (0.660- 2.940)** | 2.093 (0.878- 3.308)** | 1.895 (0.732- 3.057)** | 2.184 (0.951- 3.417)*** |
| **Size (Number of Beds)** | 0.000 (-0.002- 0.001) | 0.000 (-0.001- 0.001) | 0.000 (-0.002- 0.001) | 0.000 (-0.002- 0.001) | 0.000 (-0.002- 0.001) |
| **Staff-to-Patient Ratio** | -6.076 (-14.764- 2.613) |  | -6.032 (-14.710- 2.646) |  | -6.081 (-14.771- 2.609) |
| ***Independent Variables*** | | | | | |
| **Year** | 0.100 (-0.058- 0.258) | 0.077 (-0.072- 0.225) | 0.086 (-0.068- 0.240) | 0.090 (-0.062- 0.242) | 0.100 (-0.057- 0.258) |
| **COVID Year** | -0.347 (-1.000- 0.307) |  |  | -0.327 (-0.958- 0.304) | -0.347 (-1.000- 0.306) |
| **Dummy high degree of digitalization** | -0.340 (-1.339- 0.659) | -0.319 (-1.266- 0.629) | -0.342 (-1.323- 0.639) | -0.323 (-1.292- 0.647) | -0.340 (-1.340- 0.660) |
| **ICC** | 0.03 | 0.00 | 0.00 | 0.00 | 0.00 |
| **AIC** | 2328.653 | 2403.571 | 2329.690 | 2404.570 | 2330.634 |
| **BIC** | 2369.812 | 2440.964 | 2370.849 | 2446.118 | 2375.908 |
| *Note:* Coefficients are reported with 95% confidence intervals in parentheses. * *p* < 0.05, ** p < 0.01, *** p < 0.001.  *Note:* Variance-Inflation Factors (VIF) are reported in Appendix F. All VIF values were below the threshold of 5 (mean = 1.8), indicating no relevant multicollinearity. | | | | |  |

| **Table 5: Personnel-Expense-Ratio (PER) – HIMSS EMRAM excluding hospitals that were reclassified or upgraded to HIMSS Stage 6 or 7 during the study period Mixed-effect Model development** | | | | | |
| --- | --- | --- | --- | --- | --- |
| **Parameter** | **Null Model** | **Model 1** | **Model 2** | **Model 2.1** | **Model 3** |
| **Intercept** | 48.680 (47.411- 49.950) | 1453.156 (747.102- 2159.211)*** | 1427.283 (737.347- 2117.218)*** | 1377.509 (674.797- 2080.222)*** | 1611.171 (945.339- 2277.003)*** |
| ***Control Variables*** | | | | | |
| **Academic hospital** |  |  | 5.510 (2.389- 8.631)*** | 5.370 (2.211- 8.530)*** | 4.973 (1.942- 8.003)** |
| **Size (Number of Beds)** |  |  | -0.002 (-0.006- 0.002) | -0.001 (-0.005- 0.002) | -0.001 (-0.004- 0.003) |
| **Staff-to-Patient Ratio** |  |  |  | -0.382 (-13.257- 12.493) |  |
| ***Independent Variables*** | | | | | |
| **Year** |  | -0.695 (-1.045- -0.346)*** | -0.682 (-1.024- -0.341)*** | -0.658 (-1.006- -0.310)*** | -0.774 (-1.103- -0.444)*** |
| **COVID Year** |  |  |  |  | 2.991 (2.170- 3.812)*** |
| **Dummy high degree of digitalization** |  | 0.058 (-3.281- 3.396) | -0.135 (-3.268- 2.997) | 0.082 (-0.005- 0.002) | -0.119 (-3.241- 3.002) |
| **ICC** | 0.00 | 0.00 | 0.00 | 0.00 | 0.00 |
| **AIC** | 2820.091 | 2809.089 | 2785.223 | 2691.653 | 2739.178 |
| **BIC** | 2836.685 | 2833.980 | 2818.360 | 2728.597 | 2776.457 |
| *Note:* Coefficients are reported with 95% confidence intervals in parentheses. * *p* < 0.05, ** p < 0.01, *** p < 0.001.  *Note:* Variance-Inflation Factors (VIF) are reported in Appendix F. All VIF values were below the threshold of 5 (mean = 1.8), indicating no relevant multicollinearity. | | | | |  |

| **Table 5: Personnel-Expense-Ratio (PER) – HIMSS EMRAM excluding hospitals that were reclassified or upgraded to HIMSS Stage 6 or 7 during the study period Mixed-effect Model development** | | | | | |
| --- | --- | --- | --- | --- | --- |
| **Parameter** | **Model 3.1** | **Model 4** | **Model 4.1** | **Model 5** | **Model 5.1** |
| **Intercept** | 1565.344 (889.463- 2241.224)*** | 1427.283 (737.347- 2117.218)*** | 1377.509 (674.797- 2080.222)*** | 1611.171 (945.339- 2277.003)*** | 1565.344 (889.463- 2241.224)*** |
| ***Control Variables*** | | | | | |
| **Academic hospital** | 4.784 (1.727- 7.842)** | 5.510 (2.389- 8.631)*** | 5.370 (2.211- 8.530)*** | 4.973 (1.942- 8.003)*** | 4.784 (1.727- 7.842)** |
| **Size (Number of Beds)** | 0.000 (-0.004- 0.004) | -0.002 (-0.006- 0.002) | -0.001 (-0.005- 0.002) | -0.001 (-0.004- 0.003) | 0.000 (-0.004- 0.004) |
| **Staff-to-Patient Ratio** | -1.195 (-13.246- 10.855) |  | -0.382 (-13.257- 12.493) |  | -1.195 (-13.246- 10.855) |
| ***Independent Variables*** | | | | | |
| **Year** | -0.751 (-1.086- 0.417) | -0.682 (-1.024- -0.341)*** | -0.658 (-1.006- -0.310)*** | -0.774 (-1.103- -0.444)*** | -0.751 (-1.086- -0.417)*** |
| **COVID Year** | 3.096 (2.267- 3.924)*** |  |  | 2.991 (2.170- 3.812)*** | 3.096 (2.267- 3.924)*** |
| **Dummy high degree of digitalization** | 0.076 (-3.088- 3.239) | -0.135 (2.389- 8.631)*** | 0.082 (-3.084- 3.247) | -0.119 (-3.241- 3.002) | 0.076 (-3.088- 3.239) |
| **ICC** | 0.00 | 0.00 | 0.00 | 0.00 | 0.00 |
| **AIC** | 2643.513 | 2787.223 | 2693.654 | 2741.178 | 2645.513 |
| **BIC** | 2684.561 | 2824.501 | 2734.702 | 2782.599 | 2690.666 |
| *Note:* Coefficients are reported with 95% confidence intervals in parentheses. * *p* < 0.05, ** p < 0.01, *** p < 0.001.  *Note:* Variance-Inflation Factors (VIF) are reported in Appendix F. All VIF values were below the threshold of 5 (mean = 1.8), indicating no relevant multicollinearity. | | | | |  |

| **Table 6: Asset-Turnover-Ratio (ATR) – HIMSS EMRAM excluding hospitals that were reclassified or upgraded to HIMSS Stage 6 or 7 during the study period Mixed-effect Model development** | | | | | |
| --- | --- | --- | --- | --- | --- |
| **Parameter** | **Null Model** | **Model 1** | **Model 2** | **Model 2.1** | **Model 3** |
| **Intercept** | 0.053 (0.026- 0.080)*** | 14.281 (-13.147- 41.710) | 14.543 (-13.072- 42.159) | 14.184 (10.670- 17.698)*** | 18.071 (-9.930- 46.072) |
| ***Control Variables*** | | | | | |
| **Academic hospital** |  |  | 0.026 (-0.072- 0.123) | 0.014 (0.000- 0.028) | 0.025 (-0.072- 0.122) |
| **Size (Number of Beds)** |  |  | -0.000 (-0.000- 0.000) | 0.000 (0.000- 0.000) | 0.000 (0.000- 0.000) |
| **Staff-to-Patient Ratio** |  |  |  | 0.009 (-0.073- 0.091) |  |
| ***Independent Variables*** | | | | | |
| **Year** |  | -0.007 (-0.021- 0.007) | -0.007 (-0.021- 0.007) | -0.007 (-0.009- -0.005)*** | -0.009 (-0.023- 0.005) |
| **COVID Year** |  |  |  |  | 0.043 (-0.018- 0.104) |
| **Dummy high degree of digitalization** |  | -0.006 (-0.084- 0.072) | -0.003 (-0.082- 0.077) | 0.008 (-0.004- 0.020) | -0.003 (-0.082- 0.076) |
| **ICC** | 0.00 | 0.00 | 0.00 | 0.00 | 0.00 |
| **AIC** | 207.832 | 210.756 | 215.713 | -1902.529 | 215.779 |
| **BIC** | 224.469 | 235.711 | 248.934 | -1865.486 | 253.153 |
| *Note:* Coefficients are reported with 95% confidence intervals in parentheses. * *p* < 0.05, ** p < 0.01, *** p < 0.001.  *Note:* Variance-Inflation Factors (VIF) are reported in Appendix F. All VIF values were below the threshold of 5 (mean = 1.8), indicating no relevant multicollinearity. | | | | |  |

| **Table 6: Asset-Turnover-Ratio (ATR) – HIMSS EMRAM excluding hospitals that were reclassified or upgraded to HIMSS Stage 6 or 7 during the study period Mixed-effect Model development** | | | | | |
| --- | --- | --- | --- | --- | --- |
| **Parameter** | **Model 3.1** | **Model 4** | **Model 4.1** | **Model 5** | **Model 5.1** |
| **Intercept** | 14.142 (10.611- 17.673)*** | 14.543 (-13.072- 42.159) | 14.184 (10.670- 17.698)*** | 18.071 (-9.930- 46.072) | 14.142 (10.611- 17.673)*** |
| ***Control Variables*** | | | | | |
| **Academic hospital** | 0.014 (0.000- 0.028) | 0.026 (-0.072- 0.123) | 0.014 (0.000- 0.028) | 0.025 (-0.072-0.122) | 0.014 (0.000- 0.028) |
| **Size (Number of Beds)** | 0.000 (0.000- 0.000) | 0.000 (0.000- 0.000) | 0.000 (0.000- 0.000) | 0.000 (0.000- 0.000) | 0.000 (0.000- 0.000) |
| **Staff-to-Patient Ratio** | 0.009 (-0.073- 0.091) |  | 0.009 (-0.073- 0.091) |  | 0.009 (-0.073- 0.091) |
| ***Independent Variables*** | | | | | |
| **Year** | -0.007 (-0.009- -0.005)*** | -0.007 (-0.021-0.007) | -0.007 (-0.009- -0.005)*** | -0.009 (-0.023- 0.005) | -0.007 (-0.009- -0.005)*** |
| **COVID Year** | -0.001 (-0.007- 0.005) |  |  | 0.043 (-0.018- 0.104) | -0.001 (-0.007- 0.005) |
| **Dummy high degree of digitalization** | 0.008 (-0.004- 0.020) | -0.003 (-0.082- 0.077) | 0.008 (-0.004- 0.020) | -0.003 (-0.082- 0.076) | 0.008 (-0.004- 0.020) |
| **ICC** | 0.00 | 0.00 | 0.00 | 0.00 | 0.00 |
| **AIC** | -1900.581 | 217.713 | -1900.529 | 217.779 | -1898.581 |
| **BIC** | -1859.422 | 255.087 | -1859.370 | 259.306 | -1853.306 |
| *Note:* Coefficients are reported with 95% confidence intervals in parentheses. * *p* < 0.05, ** p < 0.01, *** p < 0.001.  *Note:* Variance-Inflation Factors (VIF) are reported in Appendix F. All VIF values were below the threshold of 5 (mean = 1.8), indicating no relevant multicollinearity. | | | | |  |

| **Table 7: Number of Patients Treated – HIMSS EMRAM excluding hospitals that were reclassified or upgraded to HIMSS Stage 6 or 7 during the study period Mixed-effect Model development** | | | | | | |
| --- | --- | --- | --- | --- | --- | --- |
| **Parameter** | **Null Model** | **Model 1** | **Model 2** | **Model 2.1** | **Model 3** | |
| **Intercept** | 133663.712 (116946.811- 150380.613)*** | 125944.005 (108707.854- 143180.157) | 28365.895 (8583.300- 48148.490)* | 51852.301 (31480.574- 72224.027)*** | 33387.635 (13225.454- 53549.816)*** | |
| ***Control Variables*** | | | | | | |
| **Academic hospital** |  |  | -52663.603 (-85342.251- -19984.955)* | -32797.714 (-65084.654- -510.774)* | -52489.643 (-84924.605- -20054.681)* | |
| **Size (Number of Beds)** |  |  | 204.946 (167.916- 241.976)*** | 201.014 (165.399- 236.629)*** | 203.500 (166.733- 240.267)*** | |
| **Staff-to-Patient Ratio** |  |  |  | -649558.549 (-854479.007- -444638.092)*** |  | |
| ***Independent Variables*** | | | | | | |
| **Year** |  | 0.001 (.) | 0.001 (.) | 0.002 (.) | 0.002 (.) | |
| **COVID Year** |  |  |  |  | -17250.746 (-32838.870- -1262.621)* | |
| **Dummy high degree of digitalization** |  | 55169.438 (10075.782- 100263.095)** | 32360.910 (4610.625- 60111.195)* | 31155.864 (4492.893- 57818.835)* | 32639.852 (5112.987- 60166.716)* | |
| **ICC** | 0.22 | 0.17 | 0.00 | 0.00 | 0.00 | |
| **AIC** | 11794.414 | 11792.795 | 11654.481 | 11497.519 | 11652.001 | |
| **BIC** | 11810.965 | 11817.621 | 11687.531 | 11534.602 | 11689.182 | |
| *Note:* Coefficients are reported with 95% confidence intervals in parentheses. * *p* < 0.05, ** p < 0.01, *** p < 0.001.  *Note:* Variance-Inflation Factors (VIF) are reported in Appendix F. All VIF values were below the threshold of 5 (mean = 1.8), indicating no relevant multicollinearity. | | | | |  | |
| **Table 7: Number of Patients Treated – HIMSS EMRAM excluding hospitals that were reclassified or upgraded to HIMSS Stage 6 or 7 during the study period Mixed-effect Model development** | | | | | | |
| **Parameter** | **Model 3.1** | **Model 4** | **Model 4.1** | **Model 5** | **Model 5.1** | |
| **Intercept** | 56406.899 (35701.545- 77112.254)*** | 28365.895 (8583.300- 48148.490)*** | 51852.301 (31480.574- 72224.027)*** | 33273.839 (14186.990- 52360.689)*** | 56406.899 (35701.545- 77112.254)*** | |
| ***Control Variables*** | | | | | | |
| **Academic hospital** | -32693.507 (-64779.877- -607.137)* | -52663.603 (-85342.251- -19984.955)** | -32797.714 (-65084.654- -510.775)* | -52755.000 (-83501.864- -22008.136)*** | -32693.507 (-64779.877- -607.137)* | |
| **Size (Number of Beds)** | 199.632 (164.224- 235.041)*** | 204.946 (167.916- 241.976)*** | 201.014 (165.399- 236.629)*** | 203.736 (168.915- 238.557)*** | 199.632 (164.224- 235.041)*** | |
| **Staff-to-Patient Ratio** | -643586.938 (-847639.120- -439534.755)*** |  | -649558.549 (-854479.006- -444638.092)*** |  | -643586.938 (-847639.120- -439534.756)*** | |
| ***Independent Variables*** | | | | | | |
| **Year** | 0.002 (.) | 0.001 (.) | 0.002 (.) | 0.002 (.) | 0.002 (.) | |
| **COVID Year** | -15902.771 (-31179.621- -625.921)* |  |  | -17080.199 (-32133.203- -2027.194)* | -15902.771 (-31179.621- -625.921)* | |
| **Dummy high degree of digitalization** | 31390.786 (4906.302- 57875.271)* | 32360.910 (4610.625- 60111.195) | 31155.864 (4492.894- 57818.834)* | 32500.686 (6444.051- 58557.320)** | 31390.786 (4906.302- 57875.271)* | |
| **ICC** | 0.00 | 0.00 | 0.00 | 0.00 | 0.00 | |
| **AIC** | 11495.354 | 11656.482 | 11499.519 | 11656.218 | 11497.354 | |
| **BIC** | 11536.557 | 11693.663 | 11540.722 | 11697.530 | 11542.677 | |
| *Note:* Coefficients are reported with 95% confidence intervals in parentheses. * *p* < 0.05, ** p < 0.01, *** p < 0.001.  *Note:* Variance-Inflation Factors (VIF) are reported in Appendix F. All VIF values were below the threshold of 5 (mean = 1.8), indicating no relevant multicollinearity. | | | | |  | |
| **Table 8: Length of Stay (LOS) – HIMSS EMRAM excluding hospitals that were reclassified or upgraded to HIMSS Stage 6 or 7 during the study period Mixed-effect Model development** | | | | | |  |
| **Parameter** | **Null Model** | **Model 1** | **Model 2** | **Model 2.1** | **Model 3** |  |
| **Intercept** | 1.171 (0.920- 1.422)*** | 75.252 (47.954- 102.551)*** | 75.351 (46.551- 104.151)*** | 159.670 (123.629- 195.710)*** | 42.385 (-31.452- 116.222) |  |
| ***Control Variables*** | | | | | |  |
| **Academic hospital** |  |  | 0.140 (-0.719- 1.000) | -0.500 (-1.320- 0.320) | 0.146 (-0.713- 1.005) |  |
| **Size (Number of Beds)** |  |  | 0.000 (0.000- 0.000) | 0.000 (0.000- 0.000) | 0.000 (0.000- 0.000) |  |
| **Staff-to-Patient Ratio** |  |  |  | 15.801 (13.892- 17.709)*** |  |  |
| ***Independent Variables*** | | | | | |  |
| **Year** |  | -0.037 (-0.050- -0.023)*** | -0.037 (-0.051- -0.023)*** | -0.079 (-0.097- -0.061)*** | -0.020 (-0.423- 0.384) |  |
| **COVID Year** |  |  |  |  | -0.049 (-0.151- 0.053) |  |
| **Dummy high degree of digitalization** |  | -0.003 (-0.405- 0.399) | -0.009 (-0.412- 0.395) | 0.026 (-0.357- 0.409) | -0.020 (-0.423- 0.384) |  |
| **ICC** | 0.92 | 0.92 | 0.92 | 0.96 | 0.92 |  |
| **AIC** | 391.878 | 369.293 | 373.186 | 246.676 | 374.274 |  |
| **BIC** | 407.050 | 392.051 | 403.530 | 280.703 | 408.411 |  |
| *Note:* Coefficients are reported with 95% confidence intervals in parentheses. * *p* < 0.05, ** p < 0.01, *** p < 0.001.  *Note:* Variance-Inflation Factors (VIF) are reported in Appendix F. All VIF values were below the threshold of 5 (mean = 1.8), indicating no relevant multicollinearity. | | | | |  |  |

| **Table 8: Length of Stay (LOS) – HIMSS EMRAM excluding hospitals that were reclassified or upgraded to HIMSS Stage 6 or 7 during the study period Mixed-effect Model development** | | | | | |
| --- | --- | --- | --- | --- | --- |
| **Parameter** | **Model 3.1** | **Model 4** | **Model 4.1** | **Model 5** | **Model 5.1** |
| **Intercept** | 118.179 (51.911- 184.447)*** | 75.375 (46.553- 104.197)*** | 159.723 (123.660- 195.785)*** | 42.411 (-31.478- 116.300) | 118.152 (51.897- 184.407)*** |
| ***Control Variables*** | | | | | |
| **Academic hospital** | -0.491 (-1.310- 0.328) | 0.136 (-0.723- 0.996) | -0.497 (-1.317- 0.323) | 0.142 (-0.718- 1.001) | -0.488 (-1.307- 0.331) |
| **Size (Number of Beds)** | 0.000 (0.000- 0.000) | 0.000 (0.000- 0.000) | 0.000 (0.000- 0.000) | 0.000 (0.000- 0.000) | 0.000 (0.000- 0.000) |
| **Staff-to-Patient Ratio** | 15.923 (14.034- 17.811)*** |  | 15.811 (13.902- 17.719)*** |  | 15.932 (14.044- 17.820)*** |
| ***Independent Variables*** | | | | | |
| **Year** | -0.058 (-0.091- -0.025)*** | -0.037 (-0.051- -0.023)*** | -0.79 (-0.097- -0.061)*** | -0.020 (-0.057- 0.016) | -0.058 (-0.091- -0.025)*** |
| **COVID Year** | -0.070 (-0.164- 0.023) |  |  | -0.049 (-0.151- 0.053) | -0.071 (-0.164- 0.023) |
| **Dummy high degree of digitalization** | 0.013 (-0.371- 0.397) | -0.008 (-0.412- 0.396) | 0.027 (-0.357- 0.410) | -0.019 (-0.426- 0.385) | 0.014 (-0.370- 0.399) |
| **ICC** | 0.96 | 0.00 | 0.00 | 0.00 | 0.00 |
| **AIC** | 246.633 | 375.414 | 248.648 | 376.503 | 248.594 |
| **BIC** | 284.440 | 409.551 | 286.455 | 414.433 | 290.182 |
| *Note:* Coefficients are reported with 95% confidence intervals in parentheses. * *p* < 0.05, ** p < 0.01, *** p < 0.001.  *Note:* Variance-Inflation Factors (VIF) are reported in Appendix F. All VIF values were below the threshold of 5 (mean = 1.8), indicating no relevant multicollinearity. | | | | |  |

| **Table 9: Absenteeism – HIMSS EMRAM excluding hospitals that were reclassified or upgraded to HIMSS Stage 6 or 7 during the study period Mixed-effect Model development** | | | | | |
| --- | --- | --- | --- | --- | --- |
| **Parameter** | **Null Model** | **Model 1** | **Model 2** | **Model 2.1** | **Model 3** |
| **Intercept** | 5.797 (5.585- 6.009)*** | -718.629 (-798.556- -638.702)*** | -722.952 (-803.313- -642.591)*** | -728.911 (-811.500- 646.322)*** | -728.038 (-810.929- -645.146)*** |
| ***Control Variables*** | | | | | |
| **Academic hospital** |  |  | -0.150 (-0.620- 0.320) | -0.330 (-0.791- 0.130) | -0.151 (-0.622- 0.320) |
| **Size (Number of Beds)** |  |  | 0.000 (-0.001- 0.000) | 0.000 (-0.001- 0.000) | 0.000 (-0.001- 0.000) |
| **Staff-to-Patient Ratio** |  |  |  | 0.471 (-2.177- 3.119) |  |
| ***Independent Variables*** | | | | | |
| **Year** |  | 0.359 (0.319- 0.398)*** | 0.361 (0.321- 0.401)*** | 0.364 (0.323- 0.405)*** | 0.363 (0.322- 0.404)*** |
| **COVID Year** |  |  |  |  | -0.056 (-0.227- 0.115) |
| **Dummy high degree of digitalization** |  | -0.112 (-0.633- 0.409) | -0.093 (-0.607- 0.422) | -0.073 (-0.532- 0.386) | -0.091 (-0.606- 0.424) |
| **ICC** | 0.00 | 0.48 | 0.47 | 0.41 | 0.47 |
| **AIC** | 1438.495 | 1312.761 | 1304.640 | 1250.524 | 1306.241 |
| **BIC** | 1455.149 | 1337.741 | 1337.896 | 1287.588 | 1343.654 |
| *Note:* Coefficients are reported with 95% confidence intervals in parentheses. * *p* < 0.05, ** p < 0.01, *** p < 0.001.  *Note:* Variance-Inflation Factors (VIF) are reported in Appendix F. All VIF values were below the threshold of 5 (mean = 1.8), indicating no relevant multicollinearity. | | | | |  |

| **Table 9: Absenteeism – HIMSS EMRAM excluding hospitals that were reclassified or upgraded to HIMSS Stage 6 or 7 during the study period Mixed-effect Model development** | | | | | |
| --- | --- | --- | --- | --- | --- |
| **Parameter** | **Model 3.1** | **Model 4** | **Model 4.1** | **Model 5** | **Model 5.1** |
| **Intercept** | -737.191 (-822.301- -652.082)*** | -722.980 (-803.293- -642.666)*** | -728.942 (-811.485- -646.398)*** | -728.056 (-810.901- -645.212)*** | -737.217 (-822.281- -652.152)*** |
| ***Control Variables*** | | | | | |
| **Academic hospital** | -0.331 (-0.792- 0.130) | -0.149 (-0.619- 0.321) | -0.330 (-0.791- 0.131) | -0.150 (-0.622- 0.321) | -0.331 (-0.792- 0.131) |
| **Size (Number of Beds)** | 0.000 (-0.001- 0.000) | 0.000 (-0.001- 0.000) | 0.000 (-0.001- 0.000) | 0.000 (-0.001- 0.000) | 0.000 (-0.001- 0.000) |
| **Staff-to-Patient Ratio** | 0.470 (-2.177- 3.117) |  | 0.472 (-2.177- 3.120) |  | 0.471 (-2.176- 3.118) |
| ***Independent Variables*** | | | | | |
| **Year** | 0.368 (0.326- 0.410)*** | 0.361 (0.321- 0.401) | 0.364 (0.323- 0.405)*** | 0.363 (0.322- 0.404)*** | 0.368 (0.326- 0.410)*** |
| **COVID Year** | -0.087 (-0.263- 0.089) |  |  | -0.055 (-0.226- 0.116) | -0.087 (-0.263- 0.089) |
| **Dummy high degree of digitalization** | -0.070 (-0.529- 0.389) | -0.093 (-0.608- 0.422) | -0.073 (-0.533- 0.386) | -0.091 (-0.606- 0.424) | -0.070 (-0.529- 0.389) |
| **ICC** | 0.38 | 0.00 | 0.00 | 0.00 | 0.00 |
| **AIC** | 1251.589 | 1306.545 | 1252.425 | 1308.148 | 1253.491 |
| **BIC** | 1292.770 | 1343.958 | 1293.605 | 1349.716 | 1298.790 |
| *Note:* Coefficients are reported with 95% confidence intervals in parentheses. * *p* < 0.05, ** p < 0.01, *** p < 0.001.  *Note:* Variance-Inflation Factors (VIF) are reported in Appendix F. All VIF values were below the threshold of 5 (mean = 1.8), indicating no relevant multicollinearity. | | | | |  |
